# ARISE: RNA-anchored shared-edge topology and hierarchical fusion for spatial multi-omics integration

**DOI:** 10.1093/bioinformatics/btag465

**Published:** 2026-06-29

**Authors:** Xiangxiang Wang, Yanchi Su, Gaoyang Hao, Meng Wang, Yunhe Wang, Xiangtao Li

**Affiliations:** School of Artificial Intelligence, Jilin University, Changchun 130012, China; School of Information Science and Technology, Northeast Normal University, Changchun 130117, China; School of Artificial Intelligence, Jilin University, Changchun 130012, China; School of Artificial Intelligence, Hebei University of Technology, Tianjin 300401, China; School of Artificial Intelligence, Hebei University of Technology, Tianjin 300401, China; School of Artificial Intelligence, Jilin University, Changchun 130012, China

## Abstract

**Motivation:**

Spatial multi-omics technologies jointly profile transcriptomes, proteins and chromatin accessibility in situ, enabling integrative analysis of tissue organization across molecular layers. However, most existing graph-based integration methods rely on independently constructed modality-specific k-nearest-neighbor graphs. When auxiliary modalities are sparse or noisy, these graphs can become topologically discordant, propagate spurious edges, weaken cross-modal alignment, and reduce spatial domain resolution.

**Results:**

We present *Anchored RNA for Integrated Spatial Embedding* (ARISE), an RNA expression anchored framework for spatial multi-omics integration. ARISE defines a shared-edge topology by intersecting RNA feature-similarity and spatial-proximity graphs, encodes auxiliary modalities on this common scaffold, and integrates them through inside-out hierarchical fusion. We further show theoretically that graph intersection minimizes false-positive edges within a broad class of *k*-of-*r* graph fusion rules, providing a principled basis for topology anchoring. Across various spatial multi-omics benchmarks spanning simulated and real datasets in bi-modal and tri-modal settings, ARISE improves spatial domain identification, cross-modal consistency, and preservation of tissue structure relative to existing methods. Furthermore, the learned representation supports biologically meaningful downstream analyses, including marker-based domain annotation, pathway enrichment, and *cis*-regulatory inference, indicating that ARISE yields a robust and interpretable framework for spatial multi-omics integration.

**Availability and implementation:**

The source code is available at https://github.com/XiangxiangWang-code/ARISE. The archived version used in this study is available at https://doi.org/10.6084/m9.figshare.32686137.v2.

## 1 Introduction

Spatial multi-omics technologies jointly profile transcriptomes, proteomes, and chromatin accessibility *in situ*, enabling integrative analysis within intact tissues. This capability has reshaped the study of cellular heterogeneity, cell–cell communication, and tissue microenvironments (Hao *et al.* 2025). By capturing multiple molecular layers at single-cell and, in some settings, subcellular resolution, spatial multi-omics provides a rich framework for investigating development, disease progression, and tissue-specific function (Zhang *et al.* 2024).

In spatial omics, graph neural networks (GNNs) have emerged as a leading modeling paradigm due to their inherent ability to capture local neighborhood structures and facilitate feature propagation across spatial graphs. SpaGCN (Hu *et al.* 2021) integrates gene expression, spatial coordinates, and histology within a graph convolutional network to identify spatial domains and detect spatially variable genes. STAGATE (Dong and Zhang 2022) uses an adaptive graph attention autoencoder to learn low-dimensional embeddings that better resolve domain boundaries. SEDR (Fu *et al.* 2021) combines a deep autoencoder with a variational graph autoencoder to jointly model expression and spatial context. GraphST (Long *et al.* 2023) further extends graph-based representation learning through self-supervised contrastive learning for clustering, multi-sample integration, and deconvolution. Collectively, these studies establish GNNs as a core paradigm for spatial representation learning and domain identification (Wang *et al.* 2023).

These advances in spatial transcriptomics have recently been extended to the integration of multiple spatial modalities. Methods such as SpatialGlue (Long *et al.* 2024) and PRAGA (Huang *et al.* 2024) use graph-based encoders to combine heterogeneous molecular signals and learn joint embeddings from spatial multi-omics data. Despite differences in architecture, however, most existing approaches continue to rely on k-nearest-neighbor (kNN) graphs to define inter-spot topology. When such graphs are constructed independently for each modality, modality-specific noise, and sparsity become embedded in the neighborhood structure itself, making the resulting topologies difficult to reconcile across modalities.

This limitation is particularly pronounced for auxiliary modalities. RNA measurements are affected by dropout, whereas chromatin-accessibility profiles are often extremely sparse and near-binary; protein measurements may also exhibit substantial platform-specific variation. As a result, modality-specific kNN graphs derived from the same tissue section can encode markedly different local relationships. Propagating information over these discordant topologies can weaken cross-modal alignment, amplify spurious edges, and reduce spatial coherence, such that nearby and biologically related spots are not necessarily mapped close to one another in the latent space.

Here, we present **A**nchored **R**NA for **I**ntegrated **S**patial **E**mbedding (**ARISE**), an RNA expression anchored framework for spatial multi-omics integration. ARISE defines a shared-edge topology by intersecting an RNA feature-similarity graph with a spatial-proximity graph, thereby retaining only edges supported by both transcriptional similarity and physical neighborhood. This topology serves as a common scaffold for all modalities, onto which auxiliary measurements are projected through graph convolutions to produce structurally aligned modality-specific embeddings. A hierarchical fusion module then integrates intra-modal and cross-modal information under spatial smoothness and reconstruction constraints to generate a unified representation that preserves local tissue organization.

Complementing its architectural framework, ARISE is firmly grounded in both theoretical and empirical analyses. Theoretically, we demonstrate that graph intersection minimizes false-positive edges within a broad class of *k*-of-*r* fusion rules, providing a principled justification for prioritizing a single high-confidence topology over the merging of multiple noisy graphs. Furthermore, we derive a stability bound linking representation drift to graph deviation, which effectively characterizes the robustness of message passing against topological perturbations. Empirically, across diverse bi-modal and tri-modal benchmarks, ARISE consistently delivers accurate clustering, improved cross-modal alignment, and faithful preservation of spatial structures, thereby facilitating robust multi-omics integration.

## 2 Materials and methods

### 2.1 Overview of ARISE

**Figure 1:**
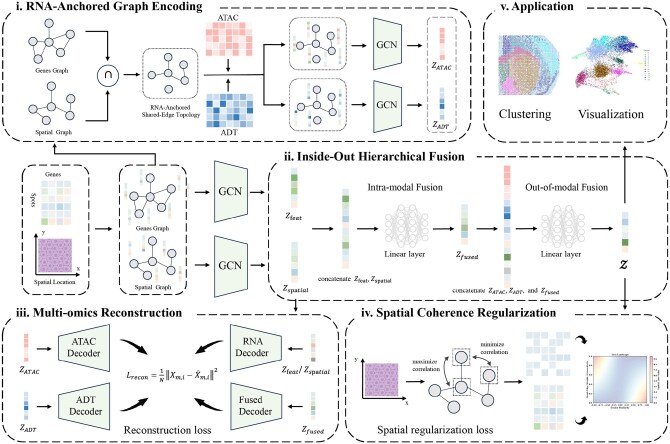
Overview of the ARISE framework. (i) RNA features and spatial coordinates are used to construct a feature-similarity graph and a spatial-proximity graph, whose edge-wise intersection defines a high-confidence RNA expression anchored shared-edge topology. Auxiliary modalities, such as ADT and ATAC, are encoded on this common scaffold via GCN-based graph convolutions, while RNA is encoded on its feature and spatial graphs to capture complementary transcriptional and spatial signals. (ii) An inside-out hierarchical fusion module first integrates the dual RNA embeddings and then progressively incorporates auxiliary modality embeddings to generate a unified latent representation. (iii, iv) Modality-specific and fused reconstruction objectives, together with spatial coherence regularization, constrain the latent space to preserve molecular information and tissue neighborhood structure. (v) The resulting representation supports downstream spatial clustering and visualization, enabling spatially coherent and biologically interpretable multi-omics integration.

As illustrated in Fig. 1, ARISE begins with modality-specific preprocessing to harmonize heterogeneous spatial multi-omics inputs before graph construction. RNA data undergo gene- and cell-level quality control, followed by selection of the top 3000 highly variable genes, total-count normalization, log1p transformation, and centering. ADT measurements are normalized independently for each spot using centered log-ratio normalization and then centered, whereas ATAC profiles are processed with TF-IDF normalization followed by latent semantic indexing. The resulting feature matrices are then integrated within an RNA expression anchored graph architecture, in which RNA defines the reference topology for cross-modal message passing. Specifically, we construct two kNN graphs on RNA, a feature-similarity graph $G_{f}=(F_{\mathrm{RNA}},A_{f})$ and a spatial-proximity graph $G_{s}=(F_{\mathrm{RNA}},A_{s})$. Their edge intersection defines a shared-edge topology


(1) $$A_{\mathrm{com}}=A_{f}\cap A_{s}$$

which retains only edges supported by both transcriptional similarity and physical adjacency. For each auxiliary modality m∈M, we instantiate $G_{m}=(F_{m},A_{\mathrm{com}})$ and learn a modality-specific embedding $Z_{m}$ using graph convolutional layers. RNA is encoded on both $A_{f}$ and $A_{s}$ to capture transcriptional similarity and local tissue context, whereas auxiliary modalities are encoded on $A_{\mathrm{com}}$ to obtain structurally aligned representations.

We then integrate these modality-specific embeddings through an inside-out hierarchical fusion strategy to obtain a unified latent representation. Fusion proceeds in two stages. We first combine the two RNA-derived embeddings to form an anchor representation that reconciles transcriptional similarity with local spatial context. We then incorporate the auxiliary modality embeddings learned on $A_{\mathrm{com}}$, so that cross-modal integration is performed on a shared-edge graph defined by RNA. This design preserves the RNA expression anchored neighborhood structure while allowing each auxiliary modality to contribute complementary molecular information through its own encoder and a lightweight fusion layer. The framework is inherently modular. Additional modalities can be seamlessly incorporated without redefining the shared-edge topology.

### 2.2 RNA expression anchored shared-edge topology

Guided by our theoretical finding that a unified graph strictly bounds false-positive connections (Theorems T1–T3, available as supplementary data at *Bioinformatics* online), we construct a shared-edge topology for spatial multi-omics integration. This effectively bypasses the vulnerabilities highlighted in prior studies, which show that cross-modal differences and noisy, modality-specific kNN graphs often lead to topology mismatch and error amplification. To anchor integration on a single, high-confidence scaffold, we designate RNA as the primary modality: transcriptomic profiles are high-dimensional and information-rich, lying downstream of chromatin and upstream of protein abundance.

Let $F_{\text{RNA}}$ denote the RNA expression matrix. We construct two adjacency matrices: a feature-similarity graph $A_{f}$ computed from cosine similarity on $F_{\text{RNA}}$, and a spatial graph $A_{s}$ computed from Euclidean distances between spatial coordinates $S\in R^{N\times 2}$. These are formally defined as:


(2) $$A_{f}^{i,j}=\{\begin{array}{cc} 1, & \text{if}\;j\in \text{TopK}\left(\text{cos}\;\;\left(f_{i}^{\mathrm{RNA}},f_{j\;}^{\mathrm{RNA}})\right)\right)\\ 0, & \text{otherwise} \end{array}$$

and


(3) $$A_{s}^{i,j}=\{\begin{array}{cc} 1, & \text{if}\;j\in \text{TopK}\left(\text{ED}\left(s_{i},s_{j}\right)\right)\\ 0, & \text{otherwise} \end{array}$$

where cos(·) denotes cosine similarity between feature vectors, and ED(·) represents the Euclidean distance between spatial coordinates.

Prior to graph intersection, we symmetrize the inherently asymmetric *k*-nearest neighbors graphs ($A_{f}$ and $A_{s}$) by establishing an undirected edge if either node is in the other’s *k*-neighborhood. This step ensures stable message passing for standard Graph Convolutional Networks (GCNs). To impose a common topology across modalities, we then intersect the symmetrized RNA feature-similarity and spatial graphs to obtain a shared adjacency via Equation (1).

This intersection acts as a conservative filter: only edges supported by both transcriptional similarity and spatial proximity are retained, reducing spurious connections and yielding a biologically grounded scaffold. We then reuse $A_{\mathrm{com}}$ for all auxiliary modalities. For each *m*, $F_{m}$ is encoded with graph convolutions on $A_{\mathrm{com}}$, which ties message passing to a consistent topology aligned with the primary modality while allowing each omics layer to contribute a complementary signal.

### 2.3 RNA-anchored multi-modal graph encoding

Building on the RNA-anchored scaffold, we encode each modality with a GNN module that (i) decouples transcriptional and spatial signals for the primary modality and (ii) reuses the shared-edge topology for auxiliary modalities to enforce a common geometry.

**Primary modality (RNA).** We construct two GCN-based encoders on the RNA feature matrix $F_{\mathrm{RNA}}$: a feature-similarity encoder on $A_{f}$ and a spatial-proximity encoder on $A_{s}$,


(4) $$Z_{\mathrm{feat}}={\tilde{A}}_{f}\text{ReLU}\left({\tilde{A}}_{f}F_{\mathrm{RNA}}W_{f}^{0}\right)W_{f}^{1}$$

and


(5) $$Z_{\mathrm{spatial}}={\tilde{A}}_{s}\text{ReLU}\left({\tilde{A}}_{s}F_{\mathrm{RNA}}W_{s}^{0}\right)W_{s}^{1}$$

where ${\tilde{A}}_{\left(\cdot \right)}$ denotes the normalized adjacency and $W_{\left(\cdot \right)}$ are learnable weights. $Z_{\mathrm{feat}}$ captures transcriptomic similarity, while $Z_{\mathrm{spatial}}$ encodes local spatial context.

**Auxiliary modalities.** For each auxiliary modality *m*, we encode $F_{\left(m\right)}$ on the shared-edge topology $A_{\mathrm{com}}$ (the RNA shared-edge graph), obtaining


(6) $$Z_{m}={\tilde{A}}_{\mathrm{com}}\text{ReLU}\left({\tilde{A}}_{\mathrm{com}}F_{m}W_{m}^{0}\right)W_{m}^{1}$$

This shared-edge encoding ties message passing to a single, biologically grounded neighborhood structure, aligning geometries across modalities while allowing each omics layer to contribute complementary signals. Together, the dual-graph RNA encoders and the shared-edge auxiliary encoders produce modality-specific embeddings that are informative, structurally consistent, and ready for the subsequent hierarchical fusion step.

As shown in Theorem T4, available as supplementary data at *Bioinformatics* online, the perturbation bound on the GNN encoder output is linear in both the number of layers *L* and the magnitude of the graph perturbation $∥E{∥}_{F}$, confirming that our encoders are stable under small changes in graph topology.

### 2.4 Hierarchical fusion of RNA and auxiliary omics representations

With the RNA dual-graph embeddings and the shared-edge embeddings from auxiliary modalities in hand, we fuse them into a single representation that preserves both intra- and cross-modal signals. We proceed inside-out: first consolidate RNA, then incorporate the auxiliaries.

We begin by concatenating the RNA embeddings from the feature-similarity and spatial graphs,


(7) $$Z_{\mathrm{concat}}^{1}=[Z_{\mathrm{feat}}⊕Z_{\mathrm{spatial}}]$$

where ⊕ is the feature concatenation operation, and project this high-dimensional vector into a unified latent space,


(8) $$Z_{\mathrm{fused}}=W_{1}Z_{\mathrm{concat}}^{1}+b_{1}$$

where $W_{1}$ and $b_{1}$ are the learnable parameters. We then append all auxiliary modality embeddings to the RNA core,


(9) $$Z_{\mathrm{concat}}^{2}=[Z_{\mathrm{fused}}⊕Z_{1}⊕Z_{2}⊕\cdots ⊕Z_{m}]$$

where ${\left\{Z_{m}\right\}}_{m=1}^{M}$ are the embeddings of *m* auxiliary modalities, and apply a second linear mapping to obtain the final multi-modal embedding,


(10) $$Z=W_{2}Z_{\mathrm{concat}}^{2}+b_{2}$$

This inside-out fusion yields a coherent latent space that integrates spatial, transcriptomic, and auxiliary omics information, providing a robust basis for downstream analyses such as cell-type annotation and microenvironment characterization (Hunter *et al.* 2021).

### 2.5 Optimization objective of ARISE

ARISE integrates the encoder-derived modality-specific graph embeddings and the fused latent representation into a composite optimization framework. This formulation simultaneously promotes spatial coherence, preserves observed molecular signals, and regularizes model complexity. Specifically, the overall loss function comprises three components: a spatial regularization term defined on the spatial graph, a reconstruction loss applied at both the modality-specific and fused levels, and standard $L_{1}$ and $L_{2}$ penalties on the network parameters.

**Spatial regularization loss.** In spatially resolved omics data, neighboring spots are more likely to share related functional states. We therefore impose a spatial smoothness constraint on the latent space, encouraging embeddings of adjacent nodes to remain similar while preserving separation from non-neighbors. This regularization improves the structural consistency of the learned representation and helps stabilize tissue-domain boundaries in the unsupervised setting.


(11) $$\begin{array}{cc} L_{\text{spatial}}=-\sum_{i,j}[A_{\text{spatial}}^{i,j}\;\text{log}\;\left(\text{sig}\left(\;\;\text{cos}(z_{i},z_{j})\right)\right) & \\ \;+\left(1-A_{\text{spatial}}^{i,j}\right)\;\text{log}\;\left(1-\text{sig}\left(\text{cos}(z_{i},z_{j})\right)\right)] & \end{array}$$

where sig(·) is the sigmoid function.

**Reconstruction loss.** To ensure that the latent representations remain faithful to the observed data, we introduce reconstruction objectives at two complementary levels. The first operates on each modality-specific embedding and preserves modality-level information, whereas the second acts on the fused embedding and constrains the integrated representation to retain the joint multi-omic signal.

For the primary modality (RNA), we decode the RNA embedding $Z_{\text{RNA}}$ with a two-layer perceptron to obtain ${\hat{F}}_{\text{RNA}}$:


(12) $${\hat{F}}_{\mathrm{RNA}}=\text{ReLU}(Z_{\mathrm{RNA}}W_{3}+b_{3})W_{4}+b_{4}$$

where $W_{3},W_{4}$, $b_{3},b_{4}$ are learnable parameters. For each auxiliary modality *m*, we reconstruct ${\hat{F}}_{m}$ from its embedding $Z_{m}$ with a modality-specific two-layer perceptron:


(13) $${\hat{F}}_{m}=\text{ReLU}(Z_{m}W_{m}^{0}+b_{m}^{0})W_{m}^{1}+b_{m}^{1}$$

where $W_{m}^{0}$, $b_{m}^{0}$, $W_{m}^{1}$, and $b_{m}^{1}$ are modality-specific learnable parameters.

The reconstruction error for each modality is defined as the discrepancy between the original features and their decoded counterparts:


(14) $$L_{m}={\left\|F_{m}-{\hat{F}}_{m}\right\|}^{2}$$

At the integrated level, we concatenate the original features across modalities,


(15) $$F_{\mathrm{fused}}=F_{1}⊕F_{2}⊕\cdots ⊕F_{M}$$

and decode the fused embedding Z with a two-layer perceptron to reconstruct ${\hat{F}}_{\text{fused}}$:


(16) $${\hat{F}}_{\mathrm{fused}}=\text{ReLU}(ZW_{5}+b_{5})W_{6}+b_{6}$$

where $W_{5},W_{6}$, $b_{5},b_{6}$ are learnable parameters. We then penalize the discrepancy between ${\hat{F}}_{\mathrm{fused}}$ and the concatenated input signal:


(17) $$L_{\text{fused}}={\left\|{\hat{F}}_{\mathrm{fused}}-F_{\mathrm{fused}}\right\|}^{2}$$

The overall reconstruction objective is defined as


(18) $$L_{\text{recon}}=\sum_{m=1}^{M}\lambda_{m}L_{m}+L_{\text{fused}}$$

where $\lambda_{m}$ controls the contribution of each modality. In this way, the model preserves modality-specific information while constraining the fused representation to remain aligned with the full multi-omic evidence.

**L1/L2 regularization.** To reduce overfitting and improve generalization, we further regularize the model parameters with combined L1 and L2 penalties:


(19) $$L_{L1,2}=\lambda_{1}\sum_{l}∥W_{l}{∥}_{1}+\lambda_{2}\sum_{l}∥W_{l}{∥}_{2}^{2}$$

where $W_{l}$ represents all learnable parameters across the entire framework, and $\lambda_{1}$ and $\lambda_{2}$ are the regularization strengths for L1 and L2.

**Total loss function.** The final training objective is a weighted combination of the three components:


(20) $$L_{\text{total}}=\alpha L_{\text{spatial}}+\beta L_{\text{recon}}+\gamma L_{L1,2}$$

where α, β, and γ are hyperparameters controlling the relative contribution of each term.

## 3 Results

### 3.1 Datasets and evaluation

We evaluated ARISE on simulated and real spatial multi-omics benchmarks spanning bi-modal and tri-modal settings. The benchmark suite comprised simulated paired RNA+ATAC spatial data, the Human Lymph Node dataset (Huang *et al.* 2024), the spatial epigenome–transcriptome mouse brain dataset (Huang *et al.* 2024), the mouse thymus stereoCITE-seq dataset (Long *et al.* 2024), Mouse Embryo E13 spatial transcriptomics dataset (Zhang *et al.* 2023), and the mouse embryo Spatial-Mux-seq dataset (Guo *et al.* 2025). Detailed dataset descriptions are provided in Section 3, available as supplementary data at *Bioinformatics* online.

We compared ARISE with representative baseline methods, including TotalVI (Gayoso *et al.* 2021), MISO (Coleman *et al.* 2025), STAGATE (Dong and Zhang 2022), PAST (Li *et al.* 2023), SpatialGlue (Long *et al.* 2024), and PRAGA (Huang *et al.* 2024). Performance was assessed using five complementary metrics. Adjusted Rand Index (ARI), Normalized Mutual Information (NMI), and Adjusted Mutual Information (AMI) quantify agreement with reference annotations when ground-truth labels are available. For unlabeled datasets, we used the Silhouette Coefficient (SC) and Davies–Bouldin index (DB) to assess cluster compactness and separation in the learned embedding space. Higher ARI, NMI, AMI, and SC indicate better performance, whereas lower DB indicates better separation. Metric definitions are provided in Section 4, available as supplementary data at *Bioinformatics* online.

### 3.2 Identification of spatial domains in simulated multi-omics data

We first assessed ARISE on simulated paired spatial RNA and ATAC data to establish performance under controlled conditions with absolute ground truth. The simulation was designed to recapitulate a challenging tissue geometry composed of five nested spatial domains. To evaluate robustness to sparsity and sample size, we generated datasets ranging from 100 to 2000 spots.

**Figure 2:**
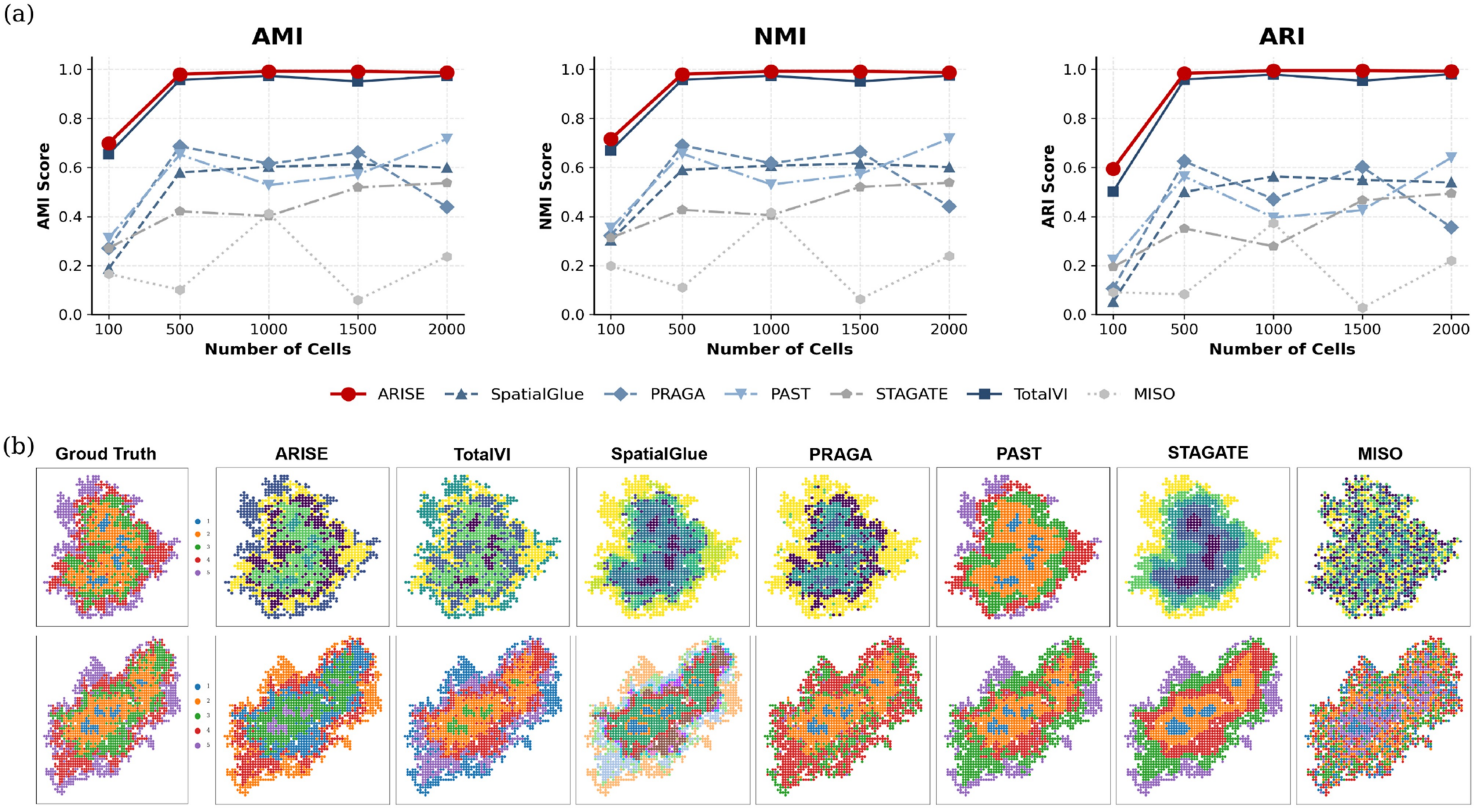
Performance on simulated spatial multi-omics (RNA+ATAC) data. (a) Quantitative comparison across simulated datasets of increasing size. (b) Recovered spatial domains on the five-layer nested benchmark.

Across all sample sizes, ARISE delivered the strongest clustering performance among the evaluated methods (Fig. 2a). For datasets with at least 500 spots, ARISE achieved near-perfect recovery of the simulated domains, with ARI, AMI, and NMI all exceeding 0.98. The advantage remained evident in the most data-limited setting. On the 100-spot benchmark, ARISE reached an ARI of 0.594, outperforming the second-best method, TotalVI, which achieved an ARI of 0.501. By contrast, most baselines, including SpatialGlue, PRAGA, and STAGATE, did not exceed an ARI of 0.65 even at larger sample sizes, indicating limited robustness to cross-modal noise.

These quantitative differences were mirrored by the reconstructed spatial maps (Fig. 2b). ARISE recovered the concentric organization of the five domains while preserving sharp and continuous boundaries across layers. TotalVI attained comparatively strong numerical scores, but its spatial assignments remained affected by salt-and-pepper noise, suggesting insufficient spatial constraint. In contrast, graph-based baselines such as SpatialGlue, PAST, and STAGATE tended to over-smooth the signal and merge fine inner layers into broader regions, whereas MISO and PRAGA disrupted the underlying spatial topology.

These simulation results indicate that the RNA expression anchored shared-edge topology of ARISE improves robustness to noisy auxiliary modalities and enables faithful recovery of fine-grained spatial structure under controlled but challenging conditions.

### 3.3 Performance on bimodal spatial multi-omics datasets

We next examined whether the advantages observed in simulation generalize to real biological data with distinct auxiliary modalities. We evaluated ARISE on RNA+ADT and RNA+ATAC datasets, which differ fundamentally in scale, sparsity, and signal structure.

**Figure 3:**
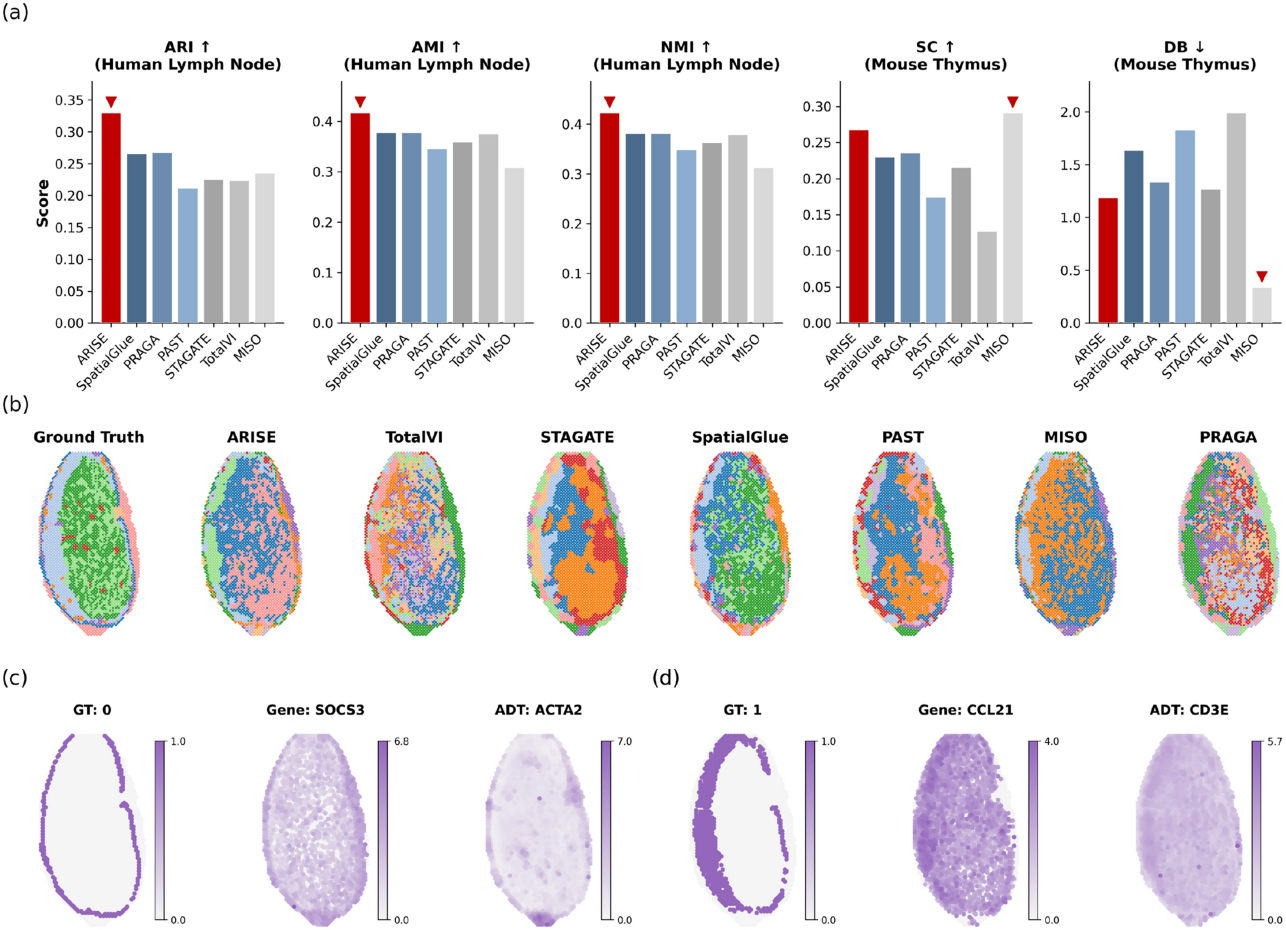
Performance on RNA+ADT datasets. (a) Quantitative comparison on Human Lymph Node (ARI, AMI, NMI) and Mouse Thymus (SC, DB). (b) Spatial domain maps for Human Lymph Node. (c) Cluster 0 corresponds to the capsule and adventitia and is marked by high ACTA2 and *SOCS3*. (d) Cluster 1 corresponds to the cortical T-cell zone and is marked by *CCL21* and CD3E.

We benchmarked ARISE on two RNA+ADT datasets: the annotated Human Lymph Node (HLN) and the unannotated Mouse Thymus. On HLN, ARISE achieved the best performance across all label-based metrics (Fig. 3a), with AMI = 0.4141, NMI = 0.4182, and ARI = 0.3427. Spatially, ARISE recovered the major lymph-node compartments (peripheral capsule, cortical region, and inner medulla) with clear inter-domain transitions, whereas TotalVI and PRAGA produced fragmented assignments and STAGATE and SpatialGlue yielded smoother but less sharply delineated domains (Fig. 3b). Bimodal marker analysis confirmed that these domains reflect genuine tissue organization: Cluster 0 localized to the peripheral rim with joint enrichment of ACTA2 protein and *SOCS3* transcript, consistent with capsular stroma (Fig. 3c), while Cluster 1 occupied the cortical T-cell zone, marked by *CCL21* expression and surface CD3E (Fig. 3d).

On the Mouse Thymus, ARISE yielded strong spatial coherence (SC = 0.2680) with well-defined structural boundaries (Fig. 1, available as supplementary data at *Bioinformatics* online). Although MISO achieved a marginally higher SC, it substantially over-smoothed the tissue, collapsing heterogeneous regions into coarse partitions. Other methods failed to resolve clear compartments or produced fragmented boundaries.

Chromatin accessibility profiles are highly sparse and often nearly binary, providing a more stringent test of cross-modal integration. We considered two benchmarks: the annotated Mouse Brain dataset and the unannotated Embryo E13 dataset.

**Figure 4:**
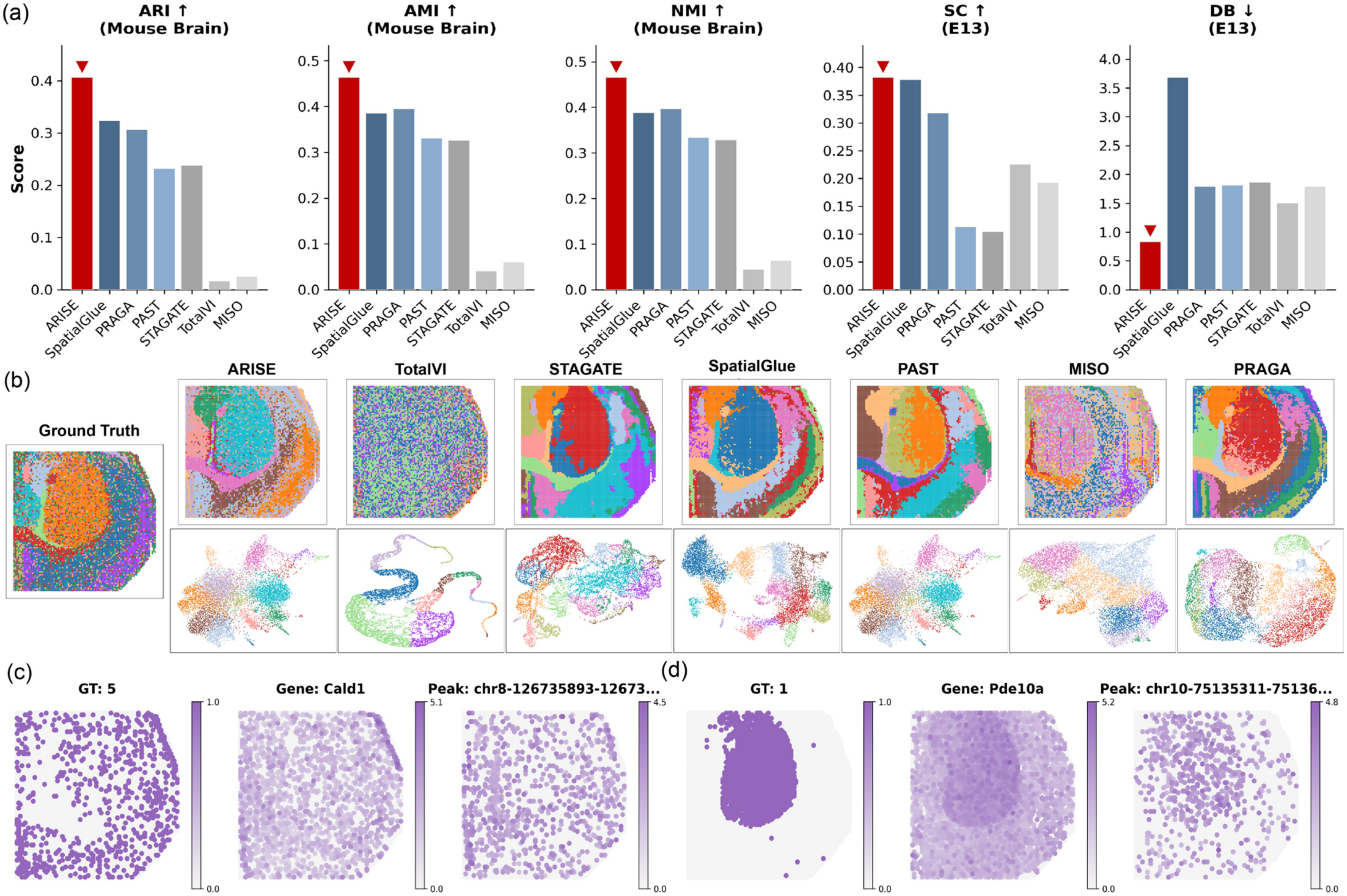
Performance on RNA+ATAC datasets. (a) Quantitative comparison on Mouse Brain (ARI, AMI, NMI) and Embryo E13 (SC, DB). (b) Spatial domain maps (top) and UMAP embeddings (bottom) for Mouse Brain, with reference annotations shown at left. (c) Cluster 5 corresponds to the striatum, marked by co-enrichment of *Pde10a* expression and its associated ATAC peak. (d) Cluster 1 corresponds to vascular and leptomeningeal cells, marked by *Cald1* expression and *cis*-regulatory chromatin accessibility.

On Mouse Brain, ARISE achieved the strongest clustering performance (Fig. 4a), while methods lacking explicit topological stabilization, including TotalVI and MISO, showed marked degradation under ATAC sparsity. ARISE faithfully recovered cortical layers and the central striatum with clear domain boundaries, whereas STAGATE and SpatialGlue showed reduced sub-regional resolution and PRAGA produced disrupted spatial assignments (Fig. 4b). The learned representation also preserved biologically meaningful chromatin–transcription coupling: Cluster 5 corresponded to vascular and leptomeningeal cells supported by concordant Cald1 expression and cis-regulatory accessibility (Fig. 4c), and Cluster 1 localized to the striatum with co-enrichment of Pde10a expression and its associated ATAC peak (Fig. 4d).

On the Embryo E13 dataset, ARISE achieved the highest SC and lowest DB among all methods and produced spatially contiguous domains recapitulating the developmental anatomy of the embryo (Fig. 2, available as supplementary data at *Bioinformatics* online). Competing methods either over-smoothed adjacent regions (TotalVI, SpatialGlue), generated fragmented assignments (STAGATE, PAST), or exhibited boundary blurring (MISO) and spatial discontinuity (PRAGA). These results demonstrate that the RNA expression anchored scaffold of ARISE yields anatomically coherent and biologically informative representations even under extreme ATAC sparsity.

### 3.4 Extension to tri-modal spatial multi-omics integration

We next examined whether ARISE extends to more highly multiplexed assays. Because the model integrates auxiliary modalities through an inside-out hierarchical fusion strategy built on a common RNA-derived scaffold, additional molecular layers can be incorporated without redefining the topological backbone.

**Figure 5:**
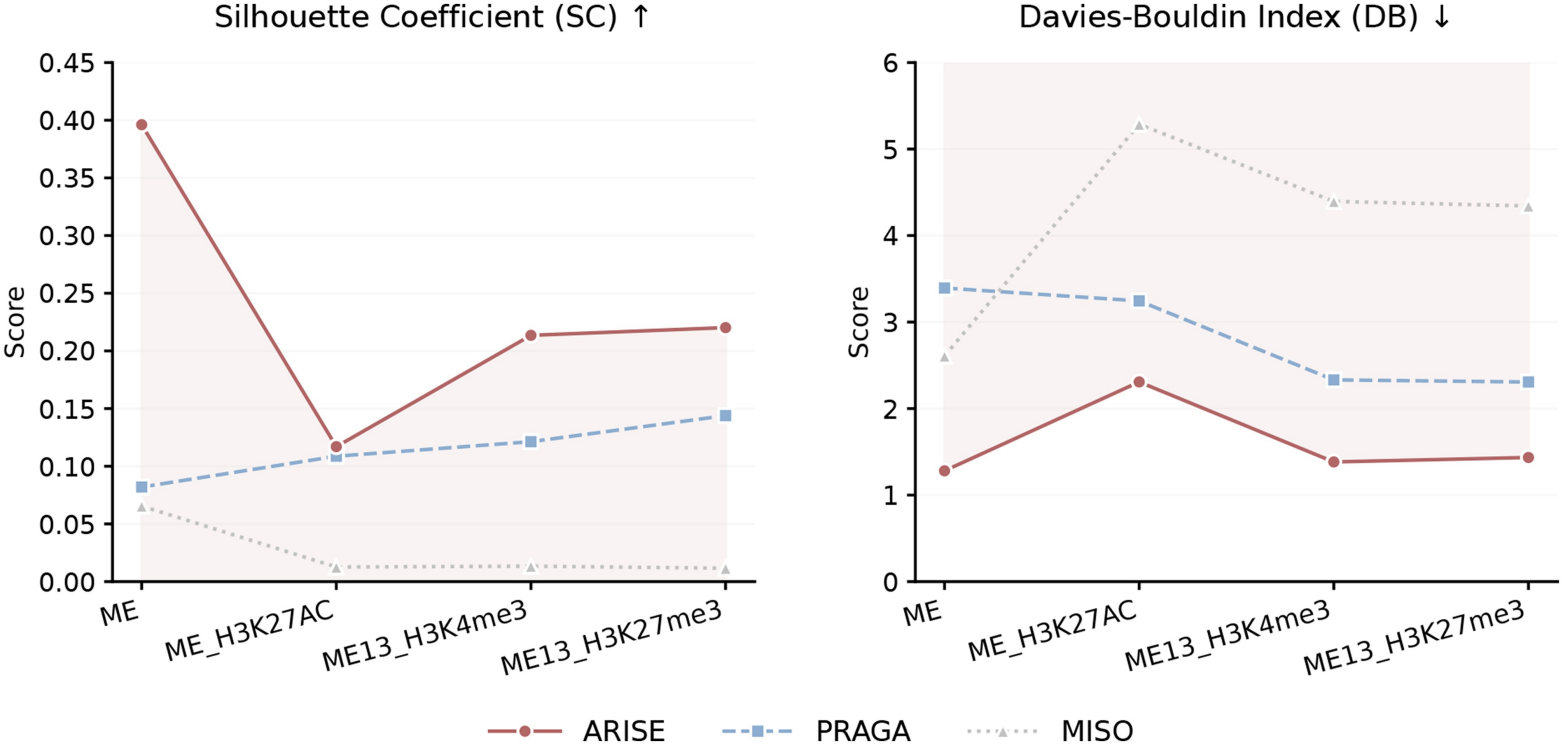
Performance on tri-modal spatial integration tasks. Quantitative comparison across four diverse Mouse Embryo datasets using the Silhouette Coefficient (SC) and Davies Bouldin (DB) index.

To evaluate scalability to tri-modal settings, we utilized four Mouse Embryo datasets. These diverse cohorts captured combinations of the spatial transcriptome with varying auxiliary layers, including chromatin accessibility (ATAC), protein abundance, and specific epigenetic marks (H3K27ac, H3K4me3, or H3K27me3). Across all four settings, ARISE yielded the highest SC and the lowest DB among the evaluated methods (Fig. 5), indicating more compact and better-separated embeddings under increasing multi-omic complexity.

The advantage was particularly evident for the histone-modification datasets, where auxiliary signals were sparse and noisy. Under these conditions, PRAGA showed unstable performance across datasets, whereas MISO approached a near-collapse in clustering cohesion, with SC values close to zero on the H3K27AC, H3K4me3, and H3K27me3 datasets. By contrast, ARISE maintained stable spatial segregation, suggesting that the RNA expression anchored scaffold provides a robust reference structure even when newly added modalities are weak or heterogeneous.

These results support the extensibility of ARISE to emerging tri-modal spatial assays and suggest that its anchoring strategy remains effective as the number and diversity of molecular layers increase.

### 3.5 Ablation and sensitivity analyses

**Figure 6:**
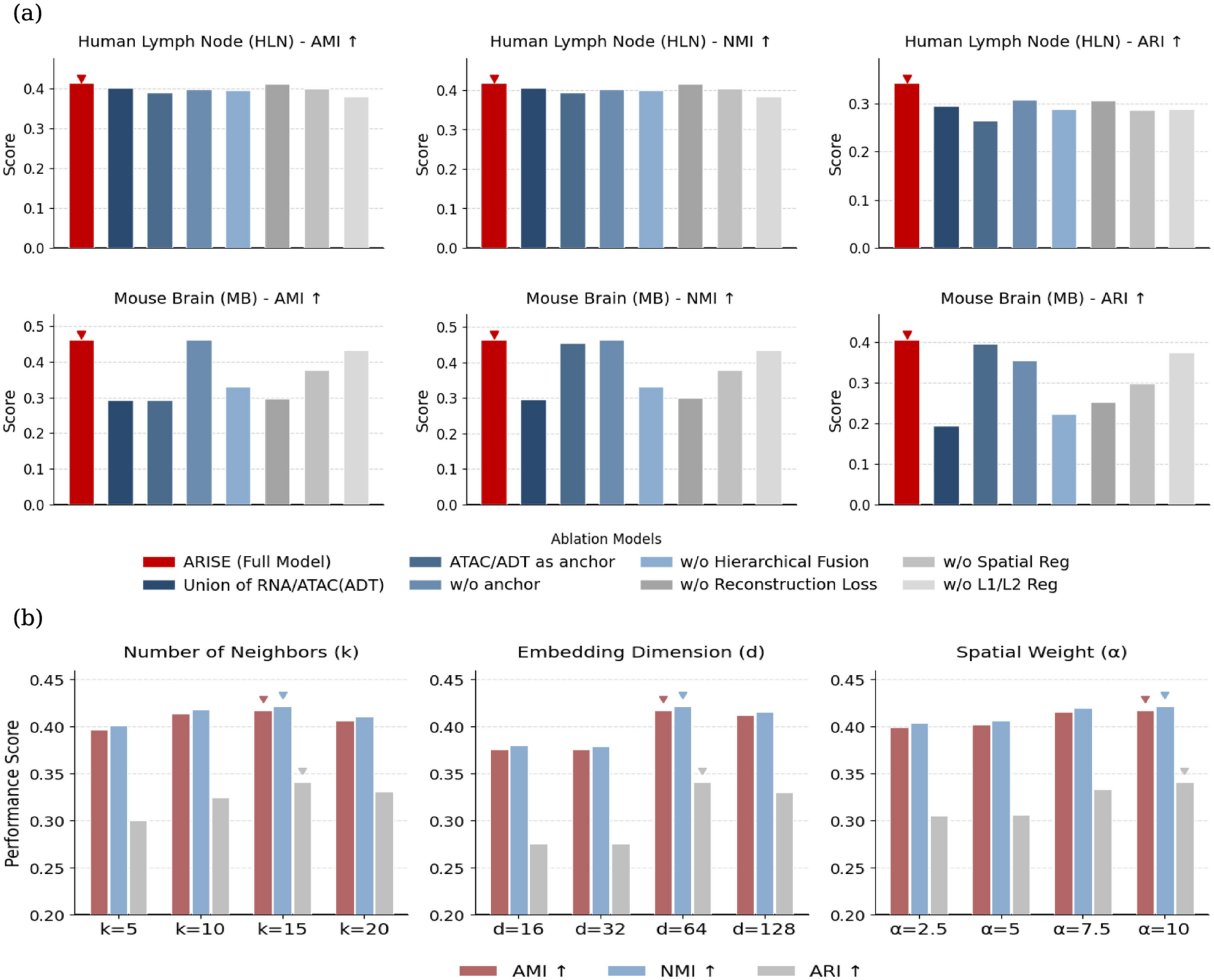
Ablation and sensitivity analyses. (a) Clustering performance of ARISE and ablation variants on the Human Lymph Node (top) and Mouse Brain (bottom) datasets. (b) Sensitivity to key hyperparameters.

To assess the contribution of each component in ARISE, we performed ablation experiments on Human Lymph Node (RNA+ADT) and Mouse Brain (RNA+ATAC) and compared the full model with representative topological and architectural variants (Fig. 6a). The full model achieved the best performance on both datasets. Replacing the RNA-derived anchor with an auxiliary-modality anchor caused the largest drop, particularly on Mouse Brain, where the sparse ATAC topology substantially reduced clustering accuracy. The Union Graph and Unanchored variants, which rely on each modality independently constructing its own *k*-nearest-neighbor graph without a shared topology, also underperformed, indicating that a shared RNA expression anchored graph provides a more reliable basis for cross-modal propagation than either edge union or modality-specific topology. Among the remaining components, removing hierarchical fusion, spatial regularization, or the reconstruction loss each impaired performance, with the strongest effect observed for spatial regularization. By contrast, L1/L2 regularization yielded smaller but consistent gains. These results suggest that the performance of ARISE depends on the joint contribution of its anchor topology, fusion mechanism, and training objective.

We further examined sensitivity to the number of neighbors *k*, embedding dimension *d*, and spatial regularization weight α on Human Lymph Node (Fig. 6b). Performance remained stable across a broad range of *k* and peaked at k=10–15. Increasing *d* improved performance up to 64, after which a slight decline was observed at 128, suggesting that a compact latent space is sufficient. Performance also improved with larger α values within the tested range and was highest at α=10, consistent with the benefit of enforcing local spatial smoothness. Overall, ARISE remained robust under moderate hyperparameter variation.

### 3.6 Biological interpretation of ARISE-derived spatial domains

To evaluate whether the ARISE latent space supports biological interpretation beyond clustering alone, we performed multi-modal marker analysis, pathway enrichment, and *cis*-regulatory analysis on the P22 Mouse Brain spatial multi-omics dataset.

**Figure 7:**
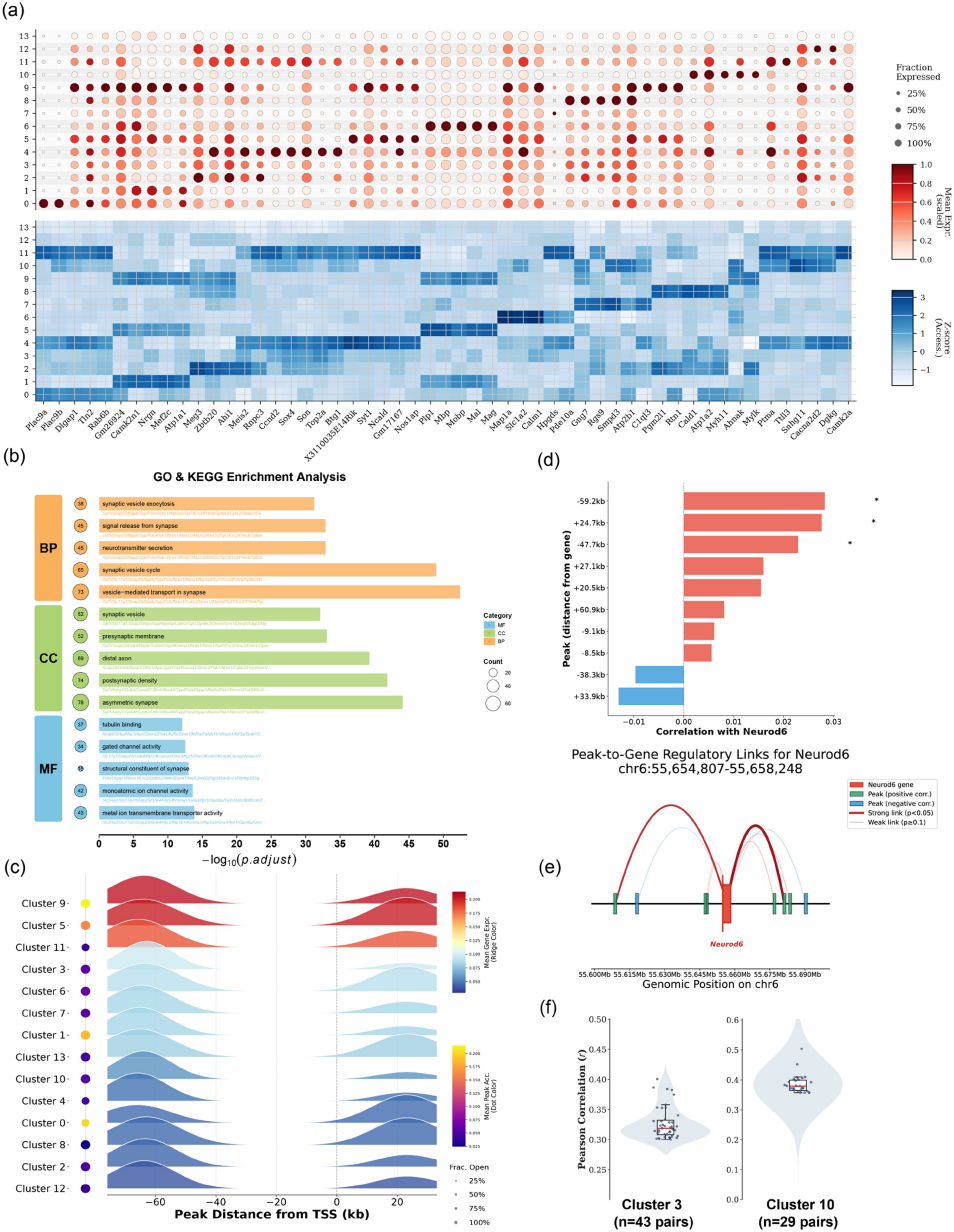
ARISE captures biologically coherent spatial domains and *cis*-regulatory states. (a) Cross-modal marker landscape showing representative gene expression (dot plot) and corresponding chromatin accessibility (heatmap) across clusters. (b) GO and KEGG enrichment analysis of cluster-specific signatures. (c) Cluster-specific ATAC accessibility ridgeline plots surrounding the *Neurod6* TSS. (d) Pearson correlation between *Neurod6* expression and candidate distal peak accessibility. (e) Inferred peak-to-gene regulatory links at the *Neurod6* locus. (f) Genome-wide peak–gene correlations, comparing regulatory coupling strength between neural progenitors (Cluster 3) and differentiated cells (Cluster 10).

A useful integrated representation should group spots not only by geometric proximity in latent space, but also by shared biological identity across molecular layers. In Fig. 7a, ARISE-derived clusters showed strong concordance between canonical transcriptomic markers and chromatin accessibility at their corresponding loci. For example, Cluster 6 displayed selective enrichment of myelin-associated genes, including *Mbp* and *Mobp*, together with increased accessibility at matched regions, consistent with mature oligodendrocytes. Likewise, Clusters 1, 7, and 13 showed coordinated enrichment of neuronal markers such as *Syt1*, *Mef2c*, and *Camk2n1*. The sharp segregation of these signals across both modalities indicates that ARISE preserves biologically coherent subpopulations while denoising the sparse ATAC layer.

We next assessed the functional relevance of these domains using KEGG pathway enrichment analysis of cluster-specific signatures (Fig. 7b). Enriched pathways included *Glutamatergic synapse*, *cAMP signaling pathway*, and *Oxytocin signaling pathway*, which are consistent with the known neurobiology of the murine brain and support the neuronal identity of the corresponding spatial regions. These results indicate that the integrated embedding retains pathway-level biological structure in addition to local domain separation.

To examine whether ARISE also supports finer-scale regulatory analysis, we focused on *Neurod6*, a transcription factor involved in neuronal differentiation. A genomic ridgeline representation of accessibility around the *Neurod6* transcription start site revealed strong cell-type specificity (Fig. 7c). Clusters with high *Neurod6* expression showed pronounced accessibility at specific distal regions, suggestive of active enhancer engagement, whereas non-expressing clusters remained comparatively inaccessible at the same loci.

We then quantified these associations by computing cell-level correlations between distal-peak accessibility and *Neurod6* expression (Fig. 7d). ARISE identified multiple peaks with strong cluster-specific accessibility and significant positive association with *Neurod6* transcription, including peaks located at −59.2kb and +24.7kb. Genomic arc visualization (Fig. 7e) further mapped these elements to a multi-enhancer regulatory architecture surrounding the *Neurod6* locus.

Finally, we extended the analysis genome-wide to examine whether peak–gene coupling varied across cell lineages. As shown in Fig. 7f, valid peak–gene pairs in Cluster 10 (VLMC1) exhibited stronger overall Pearson correlations than those in Cluster 3, annotated as subventricular zone neuroblasts (SZNBL), with median values of approximately 0.38 and 0.32, respectively. This shift is consistent with tighter regulatory coupling in differentiated structural cell types relative to the more plastic chromatin states of neural progenitors.

These analyses show that ARISE supports downstream biological interpretation at multiple levels, from marker-based annotation and pathway enrichment to candidate *cis*-regulatory inference. The learned latent space therefore functions not only as a clustering substrate, but also as a biologically grounded representation of tissue organization and regulatory state.

## 4 Discussion

ARISE addresses cross-modal topological inconsistency through a single transcriptome-anchored shared-edge topology for spatial multi-omics integration. By using RNA feature similarity together with spatial proximity to define a common scaffold for cross-modal message passing, ARISE stabilizes integration across heterogeneous modalities without reconciling multiple modality-specific graphs during representation learning. Both the theoretical analysis and the empirical results support this design, showing that graph intersection suppresses spurious edges and that the resulting framework performs robustly across simulated, bimodal, and tri-modal datasets, particularly when auxiliary modalities are sparse, noisy, or weakly structured.

Beyond improved clustering accuracy, ARISE yields biologically interpretable representations across molecular layers. In the Human Lymph Node dataset, the inferred domains aligned with known stromal and immune compartments and were supported by concordant RNA and ADT markers. In the Mouse Brain dataset, ARISE preserved coordinated patterns between gene expression and chromatin accessibility, enabling downstream analyses of marker programs, functional enrichment, and putative *cis*-regulatory interactions. The same transcriptome-anchored design, together with inside-out hierarchical fusion, also supports extension from bimodal to tri-modal settings without redefining the underlying topological structure.

The field is also witnessing the rapid development of deep learning and foundational models for multi-omics integration [e.g. scMAGCA (Wang *et al.* 2026), GARDEN (Zhang *et al.* 2026), and stDCL (Yu *et al.* 2025)]. These foundational models and ARISE function at complementary scales. The former capture generalized molecular representations via large-scale pre-training, whereas the latter explicitly models the localized, fine-grained spatial topology of individual tissue sections. A natural avenue for future work is to combine the two, for instance by using embeddings from pre-trained foundational models as initial node features within our spatial graph framework. We note that ARISE is currently limited by its reliance on RNA as the anchor modality and by its conservative shared-edge design. Future work will extend the framework to image-informed and cross-sample settings, and explore the integration of foundational-model embeddings to combine global molecular context with spatially resolved topology.

## Supplementary Material

btag465_Supplementary_Data

## Data Availability

The datasets analyzed in this study are publicly available in the following repositories. The Human lymph node, Mouse brain, and Mouse Thymus datasets can be accessed on Figshare at https://doi.org/10.6084/m9.figshare.32706993. The Mouse Embryo E13 spatial transcriptomics dataset is available in the NCBI Gene Expression Omnibus (GEO) under accession number GSE205055. The Mouse embryo Spatial-Mux-seq dataset is available in the NCBI GEO under accession number GSE263333. The source code is available at https://github.com/XiangxiangWang-code/ARISE, and published on Figshare (https://doi.org/10.6084/m9.figshare.32686137.v2).
