# Supplementary material for: ARISE: RNA-anchored shared-edge topology and hierarchical fusion for spatial multi-omics integration
Source: Bioinformatics. 2026 Jun 29;42(7):btag465. doi: 10.1093/bioinformatics/btag465 (PMC13360277; doi:10.1093/bioinformatics/btag465)
Supplement: btag465_Supplementary_Data [file btag465_supplementary_data.pdf]

Journal Title Here, YEAR, pp. 1–9
DOI added during production
Published: Date added during production
Paper

ARISE: RNA-Anchored Shared-Edge Topology and Hierarchical Fusion for Spatial Multi-Omics Integration

Xiangxiang Wang,[1,†] Yanchi Su,[2,†] Gaoyang Hao,[1] Meng Wang,[3] Yunhe Wang[3,*] and Xiangtao Li[1,*]

[1]School of Artificial Intelligence, Jilin University, 130012, Changchun, China
[2]School of Information Science and Technology, Northeast Normal University, 130117, Changchun, China
[3]School of Artificial Intelligence, Hebei University of Technology, 300401, Tianjin, China

[*]Corresponding author. wangyh082@hebut.edu.cn; lixt314@jlu.edu.cn. [†]These authors contributed equally to this work.

1 Theorems

Let $\boldsymbol{A}^* \in \{0,1\}^{N\times N}$ be the true, unknown adjacency matrix among N spots, with zero diagonal. For each modality $m \in \{1,\ldots,M\}$, let $\hat{\boldsymbol{A}}^{(m)} \in \{0,1\}^{N\times N}$ be the estimated graph. For any non-edge (i,j) of the true graph ($\boldsymbol{A}^*_{ij} = 0$), define the edge-level false positive rate (FPR) The marginal false-positive rate (FPR) for modality m is

$$p_m = \Pr\big(\hat{A}^{(m)}_{ij} = 1 \mid A^*_{ij} = 0\big) \tag{1}$$

We consider two fusion rules. Union (OR) fusion produces a graph $\hat{\boldsymbol{A}}_\vee$ that includes an edge if it appears in at least one estimated graph: $\hat{\boldsymbol{A}}_\vee = \mathbf{1}\Big\{\sum_{m=1}^{M} \hat{\boldsymbol{A}}^{(m)} \geq 1\Big\}$. Its FPR is denoted by $p_\vee$. Intersection (AND) fusion produces a graph $\hat{\boldsymbol{A}}_\wedge$ that includes an edge only if it is present in all specified source graphs; for two sources r and s, $\hat{\boldsymbol{A}}_\wedge = \hat{\boldsymbol{A}}^{(r)} \wedge \hat{\boldsymbol{A}}^{(s)}$, and its FPR is denoted by $p_\wedge$.

Theorem 1 For any two estimated graphs $\hat{\boldsymbol{A}}^{(r)}$ and $\hat{\boldsymbol{A}}^{(s)}$,

$$p_\wedge \leq \min\{p_r, p_s\}, \qquad p_\vee \geq \max_m p_m$$

Intersection never increases FPR relative to either input; union never decreases it. This inequality does not rely on cross-modality independence.

Theorem 2 If the edge errors across modalities are independent, then the FPRs of union and intersection fusion are given by

$$p_\vee = 1 - \prod_{m=1}^{M}(1-p_m), \qquad p_\wedge = \prod_{m=1}^{M} p_m.$$

Union accumulates spurious edges; intersection multiplicatively suppresses them.

Theorem 3 For r modality-specific graphs with independent false-positive indicators $\hat{A}^{(1)}_{ij}, \hat{A}^{(2)}_{ij}, \ldots, \hat{A}^{(r)}_{ij} \overset{\text{iid}}{\sim} \text{Bernoulli}(p)$, the FPR of a "$k$-of-$r$" rule that declares an edge if it appears in at least k graphs is

$$p(k) = \sum_{j=k}^{r} \binom{r}{j} p^j (1-p)^{r-j}$$

which decreases monotonically in k and is minimized at k=r (full intersection), yielding $p(r) = p^r$.

Among k-of-r strategies under the stated i.i.d. assumption, full intersection is the most conservative against false positives.

Theorems 1-3 establish that intersection minimizes FPR over all "k-of-r" rules, keeping only cross-modality-supported edges and suppressing modality noise. Hence, we use a shared-edge topology as the stable backbone, then show how our model exploits it for multi-view encoding and fusion.

Theorem 4 Consider two L-layer GNNs with identical architectures and weights $\boldsymbol{H}^{(\ell+1)} = \sigma(\tilde{\boldsymbol{A}}\boldsymbol{H}^{(\ell)}\boldsymbol{W}_\ell)$ and $\boldsymbol{H}^{(\ell+1)}_* = \sigma(\tilde{\boldsymbol{A}}^*\boldsymbol{H}^{(\ell)}_*\boldsymbol{W}_\ell)$, with σ 1-Lipschitz and $\boldsymbol{H}^{(0)} = \boldsymbol{H}^{(0)}_* = \boldsymbol{X}$, and $C_L = \prod_{i=0}^{L-1}\|\boldsymbol{W}_i\|_2$. If $\|\tilde{\boldsymbol{A}}\|_2 \leq 1$ and $\|\tilde{\boldsymbol{A}}^*\|_2 \leq 1$, then with $\boldsymbol{E} = \tilde{\boldsymbol{A}} - \tilde{\boldsymbol{A}}^*$,

$$\|\boldsymbol{H}^{(L)} - \boldsymbol{H}^{(L)}_*\|_F \leq L\,\|\boldsymbol{E}\|_F\, C_L\, \|\boldsymbol{X}\|_F \tag{2}$$

The bound in Eq. (2) is linear in the number of layers L and the magnitude of the graph perturbation $\|\boldsymbol{E}\|_F$. This confirms that our GNN encoders are stable under small changes in the graph topology.

2 Proofs

2.1 Theorem 1

$$\Big\{\hat{A}^{(r)}_{ij} = 1, \hat{A}^{(s)}_{ij} = 1\Big\} \subseteq \Big\{\hat{A}^{(r)}_{ij} = 1\Big\}, \tag{3}$$

$$\Big\{\hat{A}^{(r)}_{ij} = 1, \hat{A}^{(s)}_{ij} = 1\Big\} \subseteq \Big\{\hat{A}^{(s)}_{ij} = 1\Big\} \tag{4}$$

Therefore,

$$p_\wedge = \Pr\Big(\hat{A}^{(r)}_{ij} = 1, \hat{A}^{(s)}_{ij} = 1\Big) \leq \Pr\Big(\hat{A}^{(r)}_{ij} = 1\Big) = p_r, \quad p_\wedge \leq p_s \tag{5}$$

© The Author(s) YEAR. Copyright and licence statements to be updated by the publisher during production.

For any $k \in \{1, \dots, M\}$,

$$\left\{\hat{A}_{ij}^{(k)} = 1\right\} \subseteq \bigcup_{m=1}^{M} \left\{\hat{A}_{ij}^{(m)} = 1\right\} \tag{6}$$

Hence,

$$p_\vee = \Pr\left(\bigcup_{m=1}^{M} \left\{\hat{A}_{ij}^{(m)} = 1\right\}\right) \geq \Pr\left(\hat{A}_{ij}^{(k)} = 1\right) = p_k \tag{7}$$

which implies $p_\vee \geq \max_m p_m$. No independence is required.

2.2 Theorem 2

$$p_\vee = 1 - \Pr\left(\bigcap_{m=1}^{M} \left\{\hat{A}_{ij}^{(m)} = 0\right\}\right) \tag{8}$$

Under mutual independence,

$$\begin{aligned} \Pr\left(\bigcap_{m=1}^{M} \left\{\hat{A}_{ij}^{(m)} = 0\right\}\right) &= \prod_{m=1}^{M} \Pr\left(\hat{A}_{ij}^{(m)} = 0\right) \\ &= \prod_{m=1}^{M} (1 - p_m) \end{aligned} \tag{9}$$

so

$$p_\vee = 1 - \prod_{m=1}^{M} (1 - p_m) \tag{10}$$

For the intersection of two sources r and s, independence gives

$$\begin{aligned} p_\wedge &= \Pr\left(\hat{A}_{ij}^{(r)} = 1,\ \hat{A}_{ij}^{(s)} = 1\right) \\ &= \Pr\left(\hat{A}_{ij}^{(r)} = 1\right) \Pr\left(\hat{A}_{ij}^{(s)} = 1\right) = p_r p_s \end{aligned} \tag{11}$$

This proves the stated formulas.

2.3 Theorem 3

Assume $\hat{A}_{ij}^{(1)}, \dots, \hat{A}_{ij}^{(r)}$ are i.i.d. Bernoulli(p) for a non-edge. Define

$$S = \sum_{j=1}^{r} \mathbf{1}\left\{\hat{A}_{ij}^{(j)} = 1\right\} \tag{12}$$

Then

$$S \sim \text{Binomial}(r, p) \tag{13}$$

The FPR of the "k-of-r" rule is the binomial upper tail

$$p(k) = \Pr(S \geq k) = \sum_{j=k}^{r} \binom{r}{j} p^j (1-p)^{r-j}. \tag{14}$$

Using the pmf of S,

$$p(k) - p(k+1) = \Pr(S = k) = \binom{r}{k} p^k (1-p)^{r-k} \tag{15}$$

For $p \in (0, 1)$ the right-hand side is strictly positive, so $p(k)$ is strictly decreasing in k. Therefore the minimum is attained at $k = r$, i.e.,

$$p(r) = \Pr(S = r) = p^r \tag{16}$$

If $\hat{A}_{ij}^{(j)}$ are independent but not identically distributed with $\Pr\left(\hat{A}_{ij}^{(j)} = 1\right) = p_j$, then the AND rule corresponds to the joint event $\left\{\hat{A}_{ij}^{(1)} = \cdots = \hat{A}_{ij}^{(r)} = 1\right\}$ and

$$p(r) = \Pr\left(\hat{A}_{ij}^{(1)} = 1, \dots, \hat{A}_{ij}^{(r)} = 1\right) = \prod_{j=1}^{r} p_j \tag{17}$$

This establishes the monotonicity and the minimality of the AND rule.

2.4 Theorem 4

Consider two L-layer GNNs with identical architectures and weights, defined by the layer-wise propagation rules:

$$\boldsymbol{H}^{(\ell+1)} = \sigma(\tilde{\boldsymbol{A}} \boldsymbol{H}^{(\ell)} \boldsymbol{W}_\ell), \boldsymbol{H}_*^{(\ell+1)} = \sigma(\tilde{\boldsymbol{A}}^* \boldsymbol{H}_*^{(\ell)} \boldsymbol{W}_\ell) \tag{18}$$

with initial features

$$\boldsymbol{H}^{(0)} = \boldsymbol{H}_*^{(0)} = \boldsymbol{X} \tag{19}$$

Here, σ is a 1-Lipschitz activation function, $\boldsymbol{W}_\ell$ are the linear layer weights, and $\tilde{\boldsymbol{A}}, \tilde{\boldsymbol{A}}^*$ are the respective normalized adjacency matrices. Let

$$\boldsymbol{E} = \tilde{\boldsymbol{A}} - \tilde{\boldsymbol{A}}^* \tag{20}$$

be the perturbation matrix and

$$C_\ell := \prod_{i=0}^{\ell-1} \|\boldsymbol{W}_i\|_2 \tag{21}$$

(with $C_0 = 1$). If $\|\tilde{\boldsymbol{A}}\|_2 \leq 1$ and $\|\tilde{\boldsymbol{A}}^*\|_2 \leq 1$, then the Frobenius norm of the difference between the final layer outputs is bounded by:

$$\|\boldsymbol{H}^{(L)} - \boldsymbol{H}_*^{(L)}\|_F \leq L\, \|\boldsymbol{E}\|_2\, C_L\, \|\boldsymbol{X}\|_F \leq L\, \|\boldsymbol{E}\|_F\, C_L\, \|\boldsymbol{X}\|_F \tag{22}$$

Proof The proof proceeds in three main steps: establishing a norm bound on the forward pass, deriving a recursive inequality for the layer-wise error, and solving it via a telescoping argument.

Preliminary Lemma (Non-expansive Forward Pass). First, we show that the norm of the hidden representation is bounded. For any layer

$$\ell \geq 0, \|\boldsymbol{H}^{(\ell)}\|_F \leq C_\ell \|\boldsymbol{X}\|_F \tag{23}$$

Proof This follows by induction. The base case $\ell = 0$ is trivial:

$$\|\boldsymbol{H}^{(0)}\|_F = \|\boldsymbol{X}\|_F = C_0 \|\boldsymbol{X}\|_F \tag{24}$$

For the inductive step, assume

$$\|\boldsymbol{H}^{(\ell)}\|_F \leq C_\ell \|\boldsymbol{X}\|_F \tag{25}$$

Then,

$$\begin{aligned} \|\boldsymbol{H}^{(\ell+1)}\|_F = \|\sigma(\tilde{\boldsymbol{A}} \boldsymbol{H}^{(\ell)} \boldsymbol{W}_\ell)\|_F &\leq \|\tilde{\boldsymbol{A}} \boldsymbol{H}^{(\ell)} \boldsymbol{W}_\ell\|_F \\ &\leq \|\boldsymbol{H}^{(\ell)}\|_F \|\boldsymbol{W}_\ell\|_\mathbf{2} \end{aligned} \tag{26}$$

The first inequality uses the 1-Lipschitz property of σ, and the second uses the submultiplicativity of the Frobenius norm and the compatibility of the spectral and Frobenius norms. □

Recursive Inequality for Error. Let

$$\Delta_\ell := \|\boldsymbol{H}^{(\ell)} - \boldsymbol{H}_*^{(\ell)}\|_F \tag{27}$$

By definition, $\Delta_0 = 0$. For $\ell \geq 0$, we have:

$$\begin{aligned}
\Delta_{\ell+1} &= \|\sigma(\tilde{\boldsymbol{A}}\boldsymbol{H}^{(\ell)}\boldsymbol{W}_\ell) - \sigma(\tilde{\boldsymbol{A}}^*\boldsymbol{H}_*^{(\ell)}\boldsymbol{W}_\ell)\|_F \\
&\leq \|\tilde{\boldsymbol{A}}\boldsymbol{H}^{(\ell)}\boldsymbol{W}_\ell - \tilde{\boldsymbol{A}}^*\boldsymbol{H}_*^{(\ell)}\boldsymbol{W}_\ell\|_F \quad \text{(1-Lipschitz } \sigma) \\
&= \|(\tilde{\boldsymbol{A}} - \tilde{\boldsymbol{A}}^*)\boldsymbol{H}^{(\ell)}\boldsymbol{W}_\ell + \tilde{\boldsymbol{A}}^*(\boldsymbol{H}^{(\ell)} - \boldsymbol{H}_*^{(\ell)})\boldsymbol{W}_\ell\|_F \\
&\leq \underbrace{\|\boldsymbol{E}\boldsymbol{H}^{(\ell)}\boldsymbol{W}_\ell\|_F}_{\text{(I)}} + \underbrace{\|\tilde{\boldsymbol{A}}^*(\boldsymbol{H}^{(\ell)} - \boldsymbol{H}_*^{(\ell)})\boldsymbol{W}_\ell\|_F}_{\text{(II)}}
\end{aligned} \tag{28}$$

We bound the two terms from the inequality above separately. The first term is bounded using norm compatibility and Lemma 2.4:

$$\text{(I)} \leq \|\boldsymbol{E}\|_2\|\boldsymbol{H}^{(\ell)}\|_F\|\boldsymbol{W}_\ell\|_2 = \|\boldsymbol{E}\|_2\|\boldsymbol{H}^{(\ell)}\|_F\|\boldsymbol{W}_\ell\|_2. \tag{29}$$

The second term is bounded by

$$\text{(II)} \leq \|\tilde{\boldsymbol{A}}^*\|_2\Delta_\ell\|\boldsymbol{W}_\ell\|_2 \leq \Delta_\ell\|\boldsymbol{W}_\ell\|_2 \tag{30}$$

Combining these gives the recurrence:

$$\Delta_{\ell+1} \leq \Delta_\ell\|\boldsymbol{W}_\ell\|_2 + \|\boldsymbol{E}\|_2 C_{\ell+1}\|\boldsymbol{X}\|_F \tag{31}$$

Telescoping Sum Argument. To solve the recurrence, we introduce a scaled error term $\widehat{\Delta}_\ell := \Delta_\ell / C_\ell$, with $\widehat{\Delta}_0 = 0$. Dividing Eq. (31) by $C_{\ell+1} = C_\ell\|\boldsymbol{W}_\ell\|_2$ yields:

$$\frac{\Delta_{\ell+1}}{C_{\ell+1}} \leq \frac{\Delta_\ell}{C_\ell} + \|\boldsymbol{E}\|_2\|\boldsymbol{X}\|_F \tag{32}$$

which simplifies the recurrence in terms of the scaled error to:

$$\widehat{\Delta}_{\ell+1} \leq \widehat{\Delta}_\ell + \|\boldsymbol{E}\|_2\|\boldsymbol{X}\|_F \tag{33}$$

By unrolling this inequality from $\ell = 0$ to $L-1$, we get a telescoping sum:

$$\widehat{\Delta}_L \leq \widehat{\Delta}_0 + \sum_{\ell=0}^{L-1} (\|\boldsymbol{E}\|_2\|\boldsymbol{X}\|_F) = L\,\|\boldsymbol{E}\|_2\,\|\boldsymbol{X}\|_F \tag{34}$$

where we used the fact that $\widehat{\Delta}_0 = 0$. Finally, scaling back by substituting $\Delta_L = C_L\widehat{\Delta}_L$ gives the tighter bound:

$$\Delta_L \leq L\,C_L\,\|\boldsymbol{E}\|_2\,\|\boldsymbol{X}\|_F \tag{35}$$

The full result stated in the theorem follows from the fact that $\|\boldsymbol{E}\|_2 \leq \|\boldsymbol{E}\|_F$. This concludes the proof. □

3 Datasets

3.1 Simulated spatial RNA+ATAC datasets

We generated five simulated paired spatial RNA+ATAC datasets of increasing size (100, 500, 1 000, 1 500, and 2 000 spots) using the `scMultiSim` package (Li et al., 2025). Each dataset was simulated under a discrete five-leaf differentiation tree (`Phyla5()`), producing six ground-truth cell clusters. Gene expression (2 000 genes) and chromatin accessibility profiles were jointly generated with an ATAC-to-RNA contribution strength of 0.7, and spatial organization was imposed via an enhanced cell-cell interaction (CCI) layout. Sequencing noise was added using a UMI protocol with a mean capture rate of 10^4 and a mean sequencing depth of 5×10^4. These datasets provide controlled benchmarks with known ground-truth labels for evaluating robustness to data sparsity and varying sample size.

3.2 Human lymph node dataset

The Human Lymph Node dataset (Huang et al., 2024) is a spatial transcriptome analysis dataset derived from human lymph node sections, providing RNA sequencing data, Antibody-Derived Tag (ADT) data, spatial coordinates, and manual annotations of 3,484 sequencing spots.

3.3 Spatial epigenome transcriptome mouse brain dataset

The spatial epigenome–transcriptome mouse brain dataset (Huang et al., 2024) is collected from brain tissue sections of a juvenile (P22) mouse, providing ATAC–RNA-seq and CUT&Tag–RNA-seq data. We followed SpatialGlue to combine four datasets (one spatial ATAC–RNA-seq dataset and three spatial CUT&Tag–RNA-seq datasets) for quantitative analysis. A total of 9,215 sequencing spots were used in our quantitative experiments. While the spatial epigenome–transcriptome mouse brain dataset does not provide manually annotated labels, we utilize the clustering labels of RNA and ATAC to verify the consistency of the comprehensive encoding aggregated by our method with RNA and ATAC data, respectively.

3.4 Mouse thymus stereo-CITE-seq dataset

The mouse thymus stereo-CITE-seq dataset used in our experiments is a spatial multi-modal omics dataset from mouse thymus tissue (Long et al., 2024) after SpatialGlue processing. This dataset includes RNA sequencing data, ADT data, and the spatial location of 4697 sequencing spots.

3.5 Mouse Embryo E13 spatial transcriptomics dataset

The Mouse Embryo E13 spatial transcriptomics dataset used in our experiments is a spatial multi-modal omics dataset from Mus musculus tissue (Zhang et al., 2023). This dataset enables co-profiling of chromatin accessibility and transcriptome in spatially resolved tissue sections. After standard preprocessing, the processed data include RNA sequencing data, ATAC-seq data, and the spatial location of 2,187 sequencing spots, supporting integrative analysis of transcriptional and epigenomic regulation in a tissue context.

Mouse embryo Spatial-Mux-seq dataset

The Mouse embryo Spatial-Mux-seq dataset used in our experiments is a spatial multi-modal omics dataset from Mus musculus tissue (Guo et al., 2024). This dataset enables simultaneous spatial profiling of transcriptome, chromatin accessibility, histone modifications (H3K27me3, H3K27ac, H3K4me3), and selected proteins at tissue scale and near-cellular resolution. After standard Spatial-Mux-seq preprocessing, the processed dataset includes RNA sequencing data, multiple epigenomic modalities, protein measurements, and the spatial location of 10,000 sequencing spots.

Table 1 Summary of real spatial omics datasets used in our study

Dataset	Resolution	Modalities (Size)	#Clusters	GT	Species	Platform
Human Lymph Node (HLN)	55 μm	RNA (3,484 × 18,085), ADT (3,484 × 31)	8	Yes	Human	10x Genomics Visium
Mouse Brain Spatial Epigenome	20 μm	RNA (9,215 × 22,914), ATAC (9,215 × 121,068)	14	Yes	Mouse	Illumina NovaSeq 6000
Mouse Thymus Stereo-CITE-seq	—	RNA (4,697 × 23,622), ADT (4,697 × 51)	—	No	Mouse	Stereo-seq
Mouse Embryo E13	25 μm	RNA (2,187 × 30,849), ATAC (2,187 × 20,900)	—	No	Mouse	Illumina NovaSeq 6000
Mouse Embryo Spatial-Mux-seq	20 μm	RNA (10,000 × 48,440), ATAC (10,000 × 46,031), Protein (10,000 × 131)	—	No	Mouse	Illumina NovaSeq 6000
Mouse Embryo Spatial-Mux-seq (H3K27ac)	20 μm	RNA (10,000 × 30,849), ATAC (10,000 × 20,900), H3K27ac (8651 × 52685)	—	No	Mouse	Illumina NovaSeq 6000
Mouse Embryo E13 (H3K4me3)	20 μm	RNA (2,500 × 48,440), Protein (2,497 × 10), H3K4me3 (2,485 × 52,685)	—	No	Mouse	Illumina NovaSeq 6000
Mouse Embryo E13 (H3K27me3)	20 μm	RNA (2,500 × 48,440), Protein (2,497 × 10), H3K27me3 (2,486 × 52,685)	—	No	Mouse	Illumina NovaSeq 6000

4 Metrics

ARI (Huang et al., 2024) Adjusted Rand Index evaluates the similarity between the predicted clusters and the ground truth, adjusted for chance. It is defined as:

$$\text{ARI} = \frac{\text{RI} - \mathbb{E}[\text{RI}]}{\max(\text{RI}) - \mathbb{E}[\text{RI}]} \tag{36}$$

where RI is the Rand Index, and $\mathbb{E}[\text{RI}]$ is its expected value under random labelings.

NMI (Huang et al., 2024) Normalized Mutual Information is a symmetric measure of the agreement between two clusterings:

$$\text{NMI}(\mathcal{X}, \mathcal{Y}) = \frac{MI(\mathcal{X}, \mathcal{Y})}{\sqrt{H(\mathcal{X})H(\mathcal{Y})}} \tag{37}$$

where $MI(\cdot, \cdot)$ is mutual information and $H(\cdot)$ is entropy.

AMI (Huang et al., 2024) Adjusted Mutual Information corrects the mutual information score for chance:

$$\text{AMI} = \frac{MI - \mathbb{E}[MI]}{\max(H(\mathcal{X}), H(\mathcal{Y})) - \mathbb{E}[MI]} \tag{38}$$

where $\mathbb{E}[MI]$ is the expected mutual information between random clusterings.

SC (Halkidi et al., 2001) Silhouette Coefficient evaluates how well each object lies within its cluster:

$$\text{SC} = \frac{1}{N} \sum_{i=1}^{N} \frac{b(i) - a(i)}{\max\{a(i), b(i)\}} \tag{39}$$

where $a(i)$ is the mean intra-cluster distance and $b(i)$ is the mean nearest-cluster distance.

DB (Halkidi et al., 2001) Davies–Bouldin Index measures the average "similarity" between each cluster and its most similar one:

$$\text{DB} = \frac{1}{K} \sum_{i=1}^{K} \max_{j \neq i} \left(\frac{\sigma_i + \sigma_j}{d(c_i, c_j)} \right) \tag{40}$$

where σ_i is the average distance of all points in cluster i to its centroid c_i, and $d(c_i, c_j)$ is the distance between centroids of clusters i and j. The Silhouette Coefficient (SC) and the Davies Bouldin Index (DB) assess cluster cohesion and separation in the embedding space; higher SC and lower DB indicate better results.

5 Experimental Setup

5.1 Baseline Implementation Details

To ensure a fair, rigorous, and reproducible comparison, all baseline methods evaluated in this study were executed using the official open-source implementations released by their respective authors, without any independent reimplementation on our part. Throughout all experiments, we strictly adhered to the default hyperparameters, network architectures, and training procedures recommended in the original publications and official tutorials. For dataset-specific preprocessing, we followed the standard pipelines integrated within each baseline's codebase, thereby ensuring that every method was evaluated under its officially supported configuration.

5.2 Hyperparameter Configurations

To ensure full transparency and facilitate reproducibility of our framework, Table 2 summarizes the default hyperparameters used to train ARISE on different types of spatial multi-omics datasets.

Table 2 Summary of default hyperparameters used in ARISE.

Symbol	Dual-Modal	Tri-Modal	Simulation
k	15	15	15
d	64	64	128
α	10.0	20.0	10.0
β	25.0	2.5	2.5
γ	0.5	0.5	0.5
λ_m	1.0	1.0	1.0
lr	0.001	0.001	0.001
E	300	350	350

5.3 Clustering Procedure

To derive the final spatial domain assignments from the latent embeddings learned by ARISE, we employed the K-Means clustering algorithm. For datasets with available ground-truth annotations, the number of clusters was set equal to the number of annotated spatial domains. For unannotated datasets, the cluster count was determined according to the anatomical structures reported in the original publications or established biological prior knowledge. To ensure strict reproducibility of the downstream clustering results, the K-Means hyperparameters were kept consistent across all experiments. Specifically, the number of initializations with different centroid seeds was set to `n_init=10`, and the random seed was fixed to `random_state=42`.

5.4 Downstream Analysis Pipeline

To ensure reproducibility of the downstream analyses in Section 3.6, we provide the following methodological details.

GO and KEGG enrichment. Domain-specific differentially expressed genes (DEGs) were identified using the Wilcoxon rank-sum test in Scanpy. For the GO and KEGG enrichment analyses, gene symbols were mapped using the org.Mm.eg.db annotation package (v3.21.0, Bioconductor) based on the *Mus musculus* reference genome assembly GRCm39 (mm39), with Ensembl annotation updated to October 2024 and Gene Ontology updated to February

2025. Enrichment analysis was then performed using clusterProfiler with the hypergeometric test, and p-values were adjusted by the Benjamini–Hochberg (BH) procedure (adjusted $p < 0.05$).

Cis-regulatory analysis. Peak-to-gene (P2G) linkages were identified by searching for ATAC-seq peaks within a ± 50 kb window of target genes using the GENCODE vM34 annotation. Pearson correlations between normalized peak accessibility and gene expression across spatial spots were computed and tested with a two-sided Pearson test (SciPy, $p < 0.05$). Significant peak–gene pairs were visualized as arc diagrams using Matplotlib.

6 Result

6.1 Ablation on Feature Integration Hierarchy

To systematically validate the unique advantage of the proposed inside-out hierarchical fusion design in ARISE, we implemented and evaluated two alternative fusion orderings under identical experimental settings:

- **Outside-In Fusion:** This strategy first fuses cross-modal pairs before merging them together.
- **Parallel Fusion:** This strategy concatenates all three branch embeddings simultaneously in a single projection step.

The clustering performances of these variants are summarized in Table 3. The inside-out strategy achieves the highest ARI on both the Mouse Brain (MB) and HLN (Human Lymph Node dataset) datasets, along with the best NMI and AMI on HLN, demonstrating strong integration capability. On the MB dataset, the parallel strategy yields marginally higher NMI and AMI. This minor variance is likely because the MB dataset possesses a higher peak-feature-to-cell ratio, which slightly reduces the reliance on staged RNA consolidation.

Crucially, the inside-out design matches or surpasses the outside-in strategy across all metrics and datasets. This performance confirms our hypothesis that premature cross-modal mixing, which occurs before robust intra-modal representations are fully consolidated, is suboptimal for spatial multi-omics integration.

Table 3 Comparison of fusion ordering strategies on HLN and MB datasets.

Dataset	Metric	Inside-Out	Outside-In	Parallel
HLN	ARI	**0.3427**	0.3210	0.3378
	NMI	**0.4182**	0.4068	0.4176
	AMI	**0.4141**	0.4027	0.4135
MB	ARI	**0.4064**	0.3986	0.4054
	NMI	0.4824	0.4825	**0.4900**
	AMI	0.4804	0.4804	**0.4880**

6.2 Comprehensive Evaluation of Graph Fusion Strategies

To comprehensively examine the design space of graph fusion strategies, we systematically evaluated a broad spectrum of alternatives to our strict intersection topology, ranging from coarse to fine-grained designs:

- **Coarse strategies**: the Union variant (taking the union of edges) and the Unanchored variant (modality-specific graphs without a shared topology).
- **Fine-grained strategies**:
  – *Weighted fusion*: a soft combination $A_{\text{fused}} = \alpha A_{\text{RNA}} + (1-\alpha)A_{\text{spatial}}$, with α swept over $\{0.1, 0.3, 0.5, 0.7, 0.9\}$;
  – *Learnable attention-based fusion*: an attention mechanism that adaptively reweights edges from the two graphs during training.

Table 4 Sensitivity Analysis of Weighted Fusion (α) on HLN and MB Datasets

Dataset	Alpha (α)	ARI	NMI	AMI
HLN	0.1	0.2734	0.3938	0.3897
	0.3	0.2819	0.4044	0.4005
	0.5	0.2857	0.4041	0.4002
	0.7	0.2846	0.3963	0.3922
	0.9	0.3088	0.4170	0.4130
MB	0.1	0.2946	0.3432	0.3405
	0.3	0.2923	0.3449	0.3422
	0.5	0.2892	0.3443	0.3416
	0.7	0.2728	0.3345	0.3317
	0.9	0.2558	0.3218	0.3189

Table 5 Clustering Performance of Attention-based Fusion on HLN and MB Datasets

Dataset	ARI	NMI	AMI
HLN	0.3488	0.4205	0.4164
MB	0.3947	0.4550	0.4528

The results of these additional experiments are summarized in Supplementary Tables 4 and 5. While the learnable attention mechanism yields a marginal improvement on the HLN dataset, none of the fine-grained strategies surpass the strict intersection on the MB dataset. Furthermore, all weighted fusion variants consistently underperform across both datasets. This demonstrates that more complex soft fusion strategies do not yield consistent, across-the-board improvements. This counterintuitive finding motivates the theoretical justification detailed below.

Why strict intersection prevails. The superiority of the intersection-based design stems from two complementary perspectives:

(i) Theoretical robustness. As formally established in our theoretical analysis (see Supplementary Theorems), auxiliary modalities such as ATAC and ADT are inherently sparse and structurally noisy. Soft fusion strategies inevitably propagate a non-trivial fraction of noisy auxiliary-modality edges into the fused graph, thereby elevating the edge-level false positive rate during message passing. In contrast, the strict intersection acts as a hard biological filter: an edge is preserved only when it is simultaneously corroborated by spatial proximity *and* transcriptomic similarity, two largely independent biological signals. This dual-validation mechanism provides a strong guarantee against spurious connections that no soft fusion strategy can match.

(ii) Empirical performance. As detailed in the ablation and sensitivity analyses, the intersection-based topology maintains highly competitive and robust performance across diverse datasets. Notably, fine-grained strategies introduce additional hyperparameters or learnable parameters. While these additions might capture slight, dataset-specific variations (such as the marginal gain observed on HLN), they fail to deliver consistent advantages across multiple datasets. Instead, they increase model complexity and the risk of overfitting without a commensurate payoff.

In summary, the choice of strict intersection in ARISE is a principled design decision validated by both rigorous

theoretical analysis and extensive empirical comparison against more sophisticated alternatives.

6.3 Hyperparameter Sensitivity Analysis on RNA+ATAC Data

To assess the generalizability of our sensitivity conclusions across different modality combinations, we extended the hyperparameter sensitivity analysis to the Mouse Brain (MB) dataset (RNA+ATAC). Specifically, we examined the impact of three key hyperparameters: the number of nearest neighbors (k), the embedding dimension (d), and the spatial regularization weight (α).

As reported in Table 6, the MB dataset exhibits trends aligned with those observed on the HLN dataset (RNA+ADT): performance remains stable across a moderate parameter range and deteriorates only at extreme values. Notably, $\alpha = 10$ and $d = 128$ yield the best performance on both datasets, whereas the optimal value of k varies between them. We attribute this variation to dataset-specific differences in spatial resolution and tissue complexity rather than algorithmic instability, further confirming the robustness of ARISE across diverse multi-omics scenarios.

Table 6 Hyperparameter sensitivity analysis of ARISE on the Mouse Brain

Hyperparameter	Value	ARI ↑	NMI ↑	AMI ↑
Number of Neighbors (k)	5	0.4270	0.4917	0.4896
	10	0.4045	0.4760	0.4739
	15	**0.4102**	**0.4889**	**0.4869**
	20	0.3823	0.4567	0.4545
Embedding Dimension (d)	16	0.3479	0.3811	0.3786
	32	0.3249	0.3620	0.3593
	64	**0.3517**	**0.4271**	**0.4249**
	128	0.3968	0.4590	0.4569
Spatial Weight (α)	2.5	0.3289	0.4068	0.4044
	5.0	0.3933	0.4608	0.4586
	7.5	0.3888	0.4659	0.4637
	10.0	**0.4078**	**0.4715**	**0.4694**

6.4 Impact of Spatial Neighborhood Definitions on Embedding Stability

The construction of the spatial-proximity graph fundamentally depends on local neighborhood definitions, such as the number of nearest neighbors (k). Given that diverse tissue structures exhibit varying spatial correlation scales, it is crucial to investigate whether a fixed neighborhood parameter might be overly restrictive or introduce spurious connections across distinct anatomical domains.

To evaluate ARISE's robustness to varying spatial scales, we analyzed its clustering performance across different neighborhood sizes ($k \in \{5, 10, 15, 20\}$) on the HLN and MB datasets in Table 7. Results show highly stable performance across all values. This stability is driven by the RNA expression anchored "shared-edge topology", which constructs the final scaffold by intersecting the spatial-proximity and RNA-similarity graphs. Consequently, the RNA modality functions as a biological filter. Even when a large k inadvertently links distinct tissue domains, the intersection operation naturally prunes these spurious edges based on their transcriptional dissimilarity. This design confines message passing to highly confident pathways, effectively mitigating sensitivity to imprecise neighborhood definitions and ensuring robust integration across diverse tissue types.

Table 7 Clustering performance stability under varying k on the HLN and MB datasets.

Dataset	Neighbors (k)	ARI ↑	NMI ↑	AMI ↑
Human Lymph Node (HLN)	5	0.3970	0.4012	0.3001
	10	0.4139	0.4181	0.3248
	15	**0.4171**	**0.4212**	**0.3406**
	20	0.4062	0.4104	0.3309
Mouse Brain (MB)	5	**0.4270**	**0.4917**	**0.4896**
	10	0.4045	0.4760	0.4739
	15	0.4102	0.4889	0.4869
	20	0.3823	0.4567	0.4545

6.5 Sensitivity Analysis of Highly Variable Genes

To assess the robustness of the RNA expression anchored topology to the number of selected HVGs, we evaluated ARISE on the Human Lymph Node (HLN, RNA+ADT) and Mouse Brain (MB, RNA+ATAC) datasets with HVG counts ranging from 1,000 to 5,000. Results are reported in Table 8.

On HLN, clustering performance increases gradually with more HVGs and remains competitive throughout, with ARI ranging from 0.3131 to 0.3497. This suggests that the RNA expression anchored graph is stable across a wide range of gene set sizes when the auxiliary modality (ADT) is dense and well-measured.

On MB, performance peaks at 1,000 HVGs (ARI = 0.4521) and declines monotonically at higher values (ARI = 0.3600 at 5,000). This dataset-specific pattern is consistent with the sparse and near-binary nature of the ATAC auxiliary modality: when the anchor graph is constructed from a smaller, more focused gene set, cosine similarities are less contaminated by lowly expressed or uninformative genes, yielding a cleaner topological scaffold. Including a larger number of HVGs introduces additional transcriptional noise that propagates into the anchor graph and degrades downstream clustering.

Taken together, these results demonstrate that the RNA expression anchored topology of ARISE is generally robust to moderate variation in HVG count. The default setting of 3,000 HVGs achieves competitive performance on both datasets and provides a reasonable balance between retaining sufficient biological variance and excluding technical noise.

Table 8 Sensitivity analysis of the number of HVGs on HLN and MB datasets.

	HLN (RNA+ADT)			MB (RNA+ATAC)		
HVGs	ARI↑	NMI↑	AMI↑	ARI↑	NMI↑	AMI↑
1,000	0.3131	0.4132	0.4090	**0.4521**	**0.5268**	**0.5249**
2,000	0.3314	0.4101	0.4060	0.4201	0.4698	0.4676
3,000	0.3412	0.4195	0.4153	0.4323	0.4874	0.4853
4,000	0.3330	0.4171	0.4130	0.3894	0.4624	0.4602
5,000	**0.3497**	**0.4230**	**0.4190**	0.3600	0.4249	0.4226

6.6 Computational Efficiency Comparison

To assess the computational scalability of our framework, we measured the runtime and peak memory consumption of ARISE and compared them with those of two baseline methods, PRAGA and SpatialGlue. Detailed results across both real and simulated datasets are summarized in Tables Tables 9, 10, and 11.

Table 9 Runtime (seconds) comparison among ARISE, SpatialGlue, and PRAGA.

Dataset	ARISE (Ours)	SpatialGlue	PRAGA
Sim-100	**28.89**	39.88	30.52
Sim-500	**37.48**	39.73	49.42
Sim-1000	**37.80**	45.24	70.69
Sim-1500	**38.28**	41.67	90.81
Sim-2000	**39.21**	67.39	118.40
HLN	70.11	**17.77**	11.98
MB	242.84	**46.56**	105.80
MT	105.14	**53.37**	12.77
E13	90.43	**50.24**	9.73
ME	**572.39**	–	5493.14
ME_H3K27ac	**537.75**	–	6371.71
ME_H3K4me3	**120.51**	–	515.46
ME_H3K27me3	**249.30**	–	649.91

Table 10 Peak GPU memory usage (MB) comparison among ARISE, SpatialGlue, and PRAGA.

Dataset	ARISE (Ours)	SpatialGlue	PRAGA
Sim-100	**32.21**	65.48	67.35
Sim-500	**70.06**	69.90	105.90
Sim-1000	**117.46**	75.41	221.27
Sim-1500	**168.73**	80.96	390.90
Sim-2000	**217.61**	86.50	647.33
HLN	940.17	**100.23**	1700.07
MB	3208.99	**174.62**	11388.13
MT	1274.92	**110.55**	3022.31
E13	518.68	**88.79**	652.09
ME	**4383.69**	–	67.18
ME_H3K27ac	**2191.33**	–	69.78
ME_H3K4me3	**212.49**	–	1519.80
ME_H3K27me3	**212.58**	–	1519.80

Table 11 Peak CPU memory usage (GB) comparison among ARISE, SpatialGlue, and PRAGA.

Dataset	ARISE (Ours)	SpatialGlue	PRAGA
Sim-100	**0.65**	53.16	52.97
Sim-500	**1.28**	53.13	53.62
Sim-1000	**2.06**	53.14	54.74
Sim-1500	**2.80**	53.13	55.86
Sim-2000	**3.55**	53.13	56.90
HLN	2.63	53.13	52.97
MB	9.55	53.06	52.90
MT	99.41	53.14	52.96
E13	37.59	53.06	52.90
ME	**534.03**	–	23081.67
ME_H3K27ac	**399.24**	–	23081.67
ME_H3K4me3	**42.81**	–	57.06
ME_H3K27me3	**42.83**	–	57.27

6.7 Evaluation of Highly Variable Gene (HVG) Selection Methods

To evaluate ARISE's feature selection robustness, we analyzed how different Highly Variable Gene (HVG) selection strategies impact spatial domain characterization. While ARISE defaults to the top 3,000 dispersion-ranked HVGs, this section compares this fixed threshold against three automated or adaptive methods across the HLN and MB datasets:

- *Seurat v3 vst* (Stuart et al., 2019): variance-stabilizing transformation followed by automated top-gene ranking;
- *Scanpy cell_ranger* (Zheng et al., 2017): the default Cell Ranger pipeline implemented in Scanpy;
- *Dispersion elbow*: an adaptive cutoff determined by the elbow point on the normalized dispersion curve.

Table 12 Performance Comparison of HVG Selection Methods on HLN and MB Datasets

Dataset	Method	ARI	NMI	AMI
HLN	fixed_3000	0.3427	0.4182	0.4141
	Seurat v3 vst	0.3278	0.4156	0.4115
	Scanpy cell_ranger	0.3324	0.4063	0.4021
	Dispersion elbow	0.3038	0.3865	0.3823
MB	fixed_3000	0.4064	0.4657	0.4635
	Seurat v3 vst	0.3974	0.4764	0.4743
	Scanpy cell_rangerr	0.3923	0.4672	0.4651
	Dispersion elbow	0.3896	0.4496	0.4474

The results are summarized in Table 12. Across both datasets, the fixed 3,000-HVG strategy achieves the highest Adjusted Rand Index (ARI), scoring 0.3427 on HLN and 0.4064 on MB. While automated methods such as Seurat v3 vst yield marginally higher NMI and AMI on the MB dataset, our fixed-threshold strategy maintains highly competitive and comparable performance across all primary metrics.

Notably, the dispersion-elbow method consistently underperforms other methods across both datasets, as adaptive, elbow-point-based cutoffs are highly susceptible to technical noise in regions with low UMI counts (low sequencing depth) that are common in spatial transcriptomics. This interference destabilizes the downstream kNN graph topology, thereby empirically validating our concern that adaptive cutoffs may compromise topological stability in spatial multi-omics settings.

Why a fixed threshold is preferable in our setting. Three considerations support this design choice. First, fixed-threshold HVG selection is a widely adopted convention in spatial transcriptomics benchmarks (Long et al., 2024; Huang et al., 2024), ensuring direct comparability with prior work. Second, our sensitivity analysis demonstrates that ARISE maintains stable performance across a broad range of HVG counts (1,000–5,000), confirming that 3,000 is a robust default rather than a finely tuned value. Third, as shown above, automated alternatives do not yield consistent improvements while introducing additional sources of variability.

Table 13 Clustering performance on tri-modal spatial multi-omics datasets.

Dataset	Metric	ARISE (Ours)	PRAGA	MISO
ME	SC ↑	**0.3961**	0.0820	0.0655
	DB ↓	**1.2807**	3.3939	2.6083
ME_H3K27ac	SC ↑	**0.1170**	0.1087	0.0126
	DB ↓	**2.3082**	3.2447	5.2874
ME_H3K4me3	SC ↑	**0.2135**	0.1213	0.0134
	DB ↓	**1.3832**	2.3318	4.3948
ME_H3K27me3	SC ↑	**0.2202**	0.1439	0.0116
	DB ↓	**1.4347**	2.3072	4.3425

Table 14 Bimodal clustering performance (AMI, NMI, ARI, SC, DB) on the Human Lymph Node, Mouse Brain, Mouse Thymus, and E13 datasets.

Metrics	ARISE	SpatialGlue	PRAGA	PAST	STAGATE	TotalVI	MISO
**Human Lymph Node Dataset (RNA+ADT)**							
AMI ↑	**0.4141**	0.3780	0.3781	0.3453	0.3592	0.3752	0.3085
NMI ↑	**0.4182**	0.3820	0.3820	0.3490	0.3630	0.3790	0.3127
ARI ↑	**0.3427**	0.2660	0.2680	0.2120	0.2250	0.2240	0.2354
**Mouse Brain Dataset (RNA+ATAC)**							
AMI ↑	**0.4635**	0.3861	0.3956	0.3317	0.3264	0.0409	0.0613
NMI ↑	**0.4657**	0.3890	0.3980	0.3340	0.3290	0.0450	0.0648
ARI ↑	**0.4064**	0.3240	0.3070	0.2330	0.2390	0.0170	0.0263
**Mouse Thymus Dataset (RNA+ADT)**							
SC ↑	0.2680	0.2297	0.2358	0.1747	0.2156	0.1273	**0.2911**
DB ↓	1.1865	1.6362	1.3386	1.8292	1.2691	1.9917	**0.3389**
**E13 Dataset (RNA+ATAC)**							
SC ↑	**0.3819**	0.1914	0.3182	0.1132	0.1051	0.2259	0.1934
DB ↓	**0.8378**	1.9076	1.7944	1.8206	1.8705	1.5071	1.7979

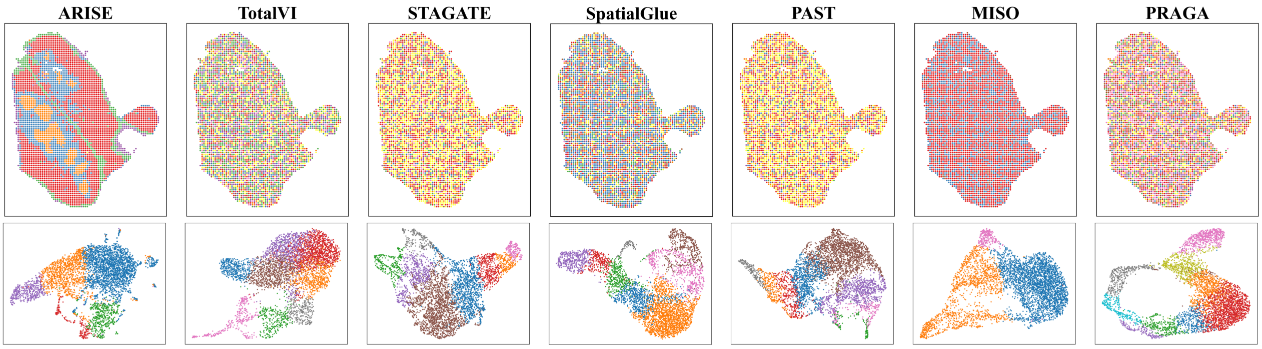

Figure 1 Clustering comparison on the Mouse Thymus dataset.

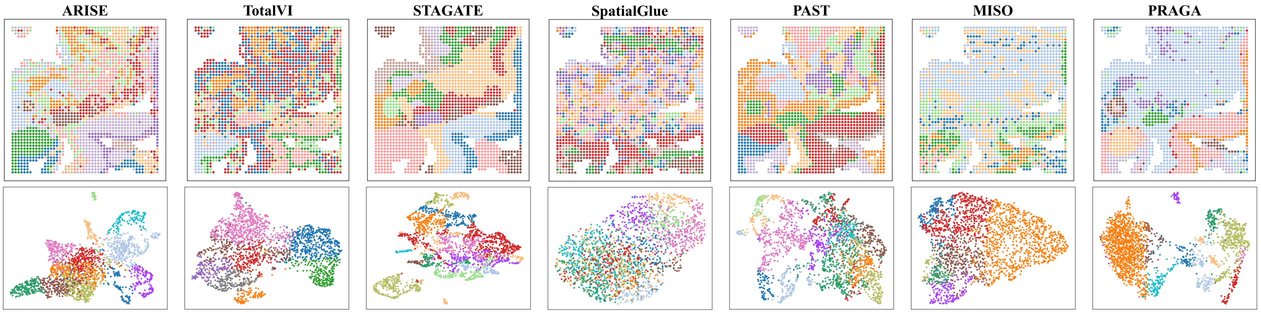

Figure 2 Clustering comparison on the Embryo E13 dataset.

References

P. Guo, L. Mao, Y. Chen, C.-N. Lee, Y. Deng, et al. Multiplexed spatial mapping of chromatin features, transcriptome, and proteins in tissues. *bioRxiv*, Sep 2024. Preprint.

M. Halkidi, Y. Batistakis, and M. Vazirgiannis. On clustering validation techniques. *Journal of Intelligent Information Systems*, 17:107–145, 2001.

X. Huang, Z. Ma, D. Meng, Y. Liu, S. Ruan, Q. Sun, X. Zheng, and Z. Qiao. Praga: Prototype-aware graph adaptive aggregation for spatial multi-modal omics analysis. In *Proceedings of the AAAI Conference on Artificial Intelligence*, 2024.

H. Li, Z. Zhang, M. Squires, and et al. scmultisim: simulation of single-cell multi-omics and spatial data guided by gene regulatory networks and cell–cell interactions. *Nature Methods*, 22:982–993, 2025.

Y. Long, K. S. Ang, R. Sethi, S. Liao, Y. Heng, L. van Olst, S. Ye, C. Zhong, H. Xu, D. Zhang, et al. Deciphering spatial domains from spatial multi-omics with spatialglue. *Nature Methods*, pages 1–10, 2024.

T. Stuart, A. Butler, P. Hoffman, et al. Comprehensive integration of single-cell data. *Cell*, 177:1888–1902.e21, 2019.

D. Zhang, Y. Deng, P. Kukanja, E. Agirre, M. Bartosovic, M. Dong, C. Ma, S. Ma, G. Su, S. Bao, et al. Spatial epigenome-transcriptome co-profiling of mammalian tissues. *Nature*, 616 (7955):113–122, 2023.

G. Zheng, J. Terry, P. Belgrader, et al. Massively parallel digital transcriptional profiling of single cells. *Nature Communications*, 8:14049, 2017. doi: 10.1038/ncomms14049.
